# The endoplasmic reticulum chaperone PfGRP170 is essential for asexual development and is linked to stress response in malaria parasites

**DOI:** 10.1111/cmi.13042

**Published:** 2019-06-06

**Authors:** Heather M. Kudyba, David W. Cobb, Manuel A. Fierro, Anat Florentin, Dragan Ljolje, Balwan Singh, Naomi W. Lucchi, Vasant Muralidharan

**Affiliations:** ^1^ Center for Tropical and Emerging Global Diseases University of Georgia Athens Georgia; ^2^ Department of Cellular Biology University of Georgia Athens Georgia; ^3^ Malaria Branch and Division of Parasitic Diseases and Malaria Centers for Disease Control and Prevention Atlanta Georgia

**Keywords:** endoplasmic reticulum, malaria, parasitology, *Plasmodium falciparum*

## Abstract

The vast majority of malaria mortality is attributed to one parasite species: Plasmodium falciparum. Asexual replication of the parasite within the red blood cell is responsible for the pathology of the disease. In *Plasmodium*, the endoplasmic reticulum (ER) is a central hub for protein folding and trafficking as well as stress response pathways. In this study, we tested the role of an uncharacterised ER protein, PfGRP170, in regulating these key functions by generating conditional mutants. Our data show that PfGRP170 localises to the ER and is essential for asexual growth, specifically required for proper development of schizonts. PfGRP170 is essential for surviving heat shock, suggesting a critical role in cellular stress response. The data demonstrate that PfGRP170 interacts with the *Plasmodium* orthologue of the ER chaperone, BiP. Finally, we found that loss of PfGRP170 function leads to the activation of the *Plasmodium* eIF2α kinase, PK4, suggesting a specific role for this protein in this parasite stress response pathway.

## 1 INTRODUCTION

Malaria is a deadly parasitic disease that causes over 212 million cases and nearly 430,000 deaths each year, primarily in children under the age of five (World Health Organization, 2017). The deadliest human malaria parasite, Plasmodium falciparum, infects individuals inhabiting subtropical and tropical regions. These are some of the most impoverished regions of the world, making diagnosis and treatment challenging. Moreover, the parasite has evolved resistance to all clinically available drugs, highlighting an important need for uncovering proteins that are essential to the biology of this parasite (Amaratunga, Witkowski, Khim, Menard, & Fairhurst, 2014; Dondorp et al., 2009; Mita et al., 2011; Roper et al., 2004; Wootton et al., 2002). Malaria is associated with a wide array of clinical symptoms, such as fever, chills, nausea, renal failure, pulmonary distress, cerebral malaria, and cardiac complications. It is the asexual replication of the parasite within the red blood cell (RBC) that is responsible for the pathology of the disease (Miller, Baruch, Marsh, & Doumbo, 2002).

In P. falciparum, the endoplasmic reticulum (ER) is a uniquely complex, poorly understood organelle. In fact, recent data suggest that ER proteins play a major role in resistance to the frontline antimalarial, artemisinin (Mok et al., 2015; Rocamora et al., 2018; Zhang et al., 2017). It is in this organelle that a variety of essential cellular functions occur, including protein trafficking, cellular signalling, and activation of stress response pathways (Boddey et al., 2013; Boddey & Cowman, 2013; Deponte et al., 2012; Russo et al., 2010; Tonkin, Kalanon, & McFadden, 2008; Walter & David, 2011; Zhang et al., 2012). Compared with other eukaryotes, the molecular mechanisms involved in these essential processes in *Plasmodium* remain poorly understood. Therefore, it is imperative to uncover proteins that regulate and maintain ER biology. One group of proteins likely governing many of these processes are ER chaperones (Araki & Nagata, 2011; Gidalevitz, Stevens, & Argon, 2013; Hotamisligil, 2010; Rutkowski & Hegde, 2010; Wang et al., 2014; Xu, Bailly‐Maitre, & Reed, 2005).Very little is known about the roles that ER chaperones play in *Plasmodium*, many of them defined merely based on sequence homology to other organisms. The *Plasmodium* genome encodes a relatively reduced repertoire of predicted ER chaperones, but it is predicted to contain two members of the conserved ER HSP70 chaperone complex, GRP78 (or BiP) and a putative HSP110 (PfGRP170 or PfHSP70‐y; Pavithra, Kumar, & Tatu, 2007; Shonhai, Boshoff, & Blatch, 2007). GRP170, in other eukaryotes, serves as nucleotide exchange factor for BiP (Andreasson, Rampelt, Fiaux, Druffel‐Augustin, & Bukau, 2010; de Keyzer, Steel, Hale, Humphries, & Stirling, 2009). Additionally, GRP170 has been reported to have holdase activity and can bind unfolded substrates independent of ATP or BiP (Behnke & Hendershot, 2014; Buck et al., 2013; Park et al., 2003).

In this study, we used a conditional auto‐inhibition strategy to generate conditional mutants for the putative ER chaperone, PfGRP170 (PF3D7_1344200; Beck, Muralidharan, Oksman, & Goldberg, 2014; Florentin et al., 2017; Muralidharan, Oksman, Pal, Lindquist, & Goldberg, 2012). Using these conditional mutants, we localised PfGRP170 to the parasite ER and show that unlike its orthologs in other eukaryotes, PfGRP170 is essential for parasite survival. Detailed life cycle analysis revealed that inhibition of PfGRP170 results in parasite death in early schizogony. The protein is required for surviving a brief heat shock, suggesting that PfGRP170 is essential during febrile episodes in the host. We show that despite a predicted transit peptide, PfGRP170 is not essential for protein trafficking to the apicoplast. Trafficking experiments using antibodies for two PEXEL negative exported proteins and one protein containing a Plasmodium Export Element indicate that PfGRP170 is unlikely to be involved in protein export. Using a combination of mass spectroscopy approaches, we identified potential interactors. Moreover, we demonstrate here that PfGRP170 interacts with the *Plasmodium* homologue of BiP (PF3D7_0917900) suggesting a conserved HSP70 ER chaperone complex. Finally, we show that conditional inhibition of PfGRP170 leads to the activation of the only known ER stress response pathway in Plasmodium, the PK4 pathway (Zhang et al., 2012; Zhang et al., 2017).

## 2 RESULTS

### 2.1 PF3D7_1344200 is a putative GRP170 in P. falciparum

**Figure 1:**
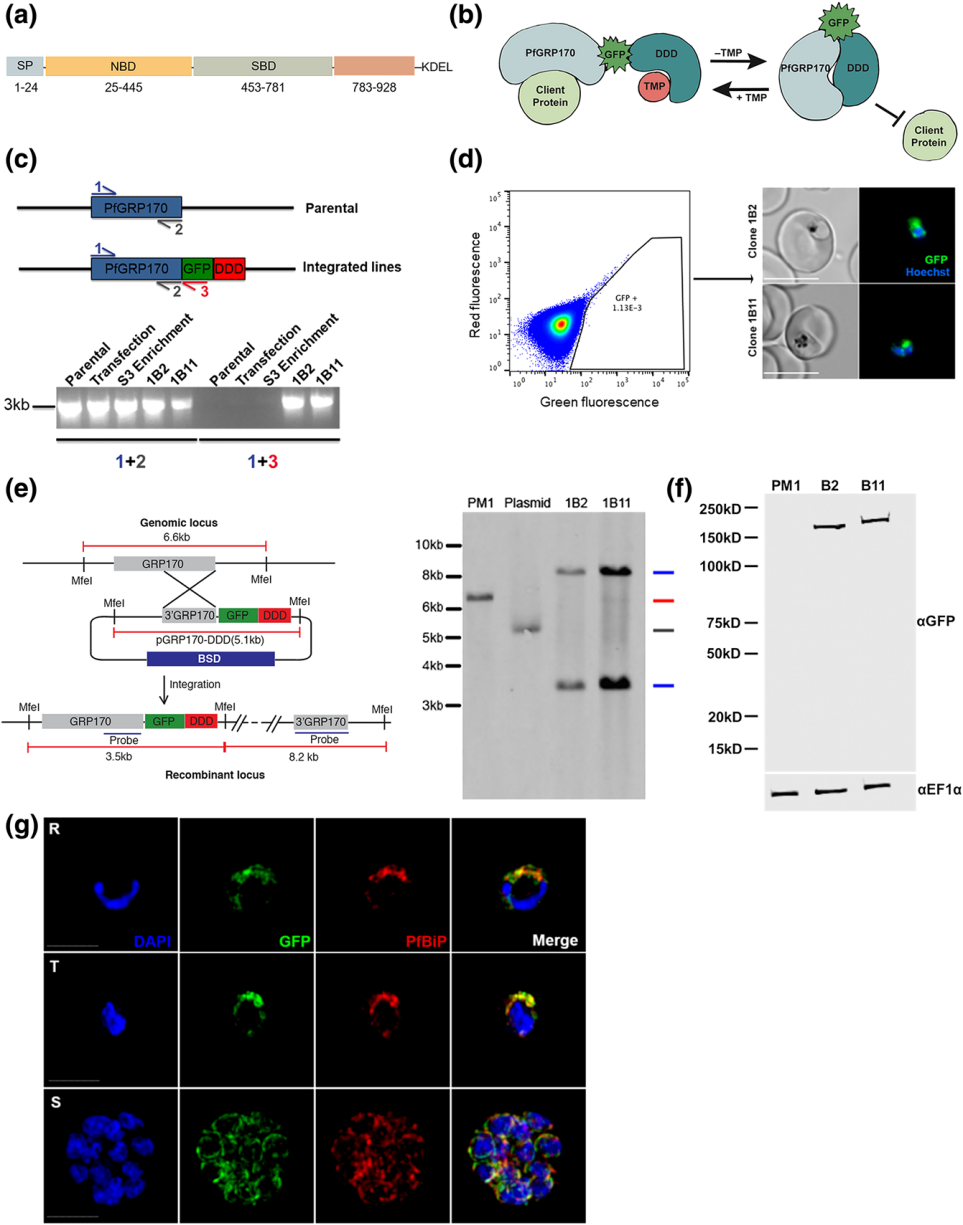
Generation of PfGRP170‐GFP‐DD Parasites. (a) Schematic detailing the putative domain boundaries of PfGRP170 (PF3D7_1344200) based on the yeast homologue, Lhs1: signal peptide (SP), nucleotide binding domain (NBD), substrate‐binding domain (SBD), extended C‐terminus region (783–928), and an ER retention signal (KDEL). (b) Schematic diagram demonstrating the conditional inhibition of PfGRP170. Conditional inhibition of PfGRP170 is achieved by the removal of trimethoprim (TMP), which results in the unfolding of the destabilisation (DDD). The chaperone recognises and binds the unfolded DDD and is inhibited from interacting with client proteins. (c) (Top) Schematic diagram of the PfGRP170 locus in the parental line (PM1KO) and the modified locus where PfGRP170 is endogenously tagged with GFP and DDD. Primers used for integration test and control PCR are indicated by arrows. The relative positions of Primer 1 (blue) and Primer 2 (grey) on the PfGRP170 locus are shown. These two primers will amplify PfGRP170 in parental and transfected parasites. Primer 3 (Red) recognises the GFP sequence. Primers 1 and 3 were used to screen for proper integration into the PfGRP170 locus. (Bottom) PCR integration test and control PCRs on gDNA isolated from the PM1KO (parental), the original transfection of the pPfGRP170‐GFP‐DDD plasmid after three rounds of blasticidin (BSD) drug selection (transfection), the PfGRP170‐GFP‐DDD transfected parasite lines after two rounds of enrichment for GFP positive cells (S3 enrichment), and PfGRP170‐GFP‐DDD clones 1B2 and 1B11 after MoFlo XDP flow sorting. The first five lanes are control PCRs using primers to amplify the PfGRP170 locus. The last five lanes are integration PCRs that only amplify if the GFP‐DDD has been integrated into the genome. (d) (Left) MoFlo XDP flow data demonstrating the percentage of GFP positive parasites in transfected PfGRP170‐GFP‐DDD parasites following three rounds of drug selection with blasticidin (BSD) and two rounds of enrichment with an S3 cell sorter. Using the MoFlo, single GFP positive cells were cloned into a 96‐well plate. Two clones, 1B2 and 1B11, were isolated using this method. (Right) 1B2 and 1B11 parasites were observed using live fluorescence microscopy. (e) Southern blot analysis of PfGRP170‐GFP‐DDD clones 1B2 and 1B11, PM1KO (parental control), and the PfGRP170‐GDB plasmid is shown. Mfe1 restriction sites, the probe used to detect the DNA fragments, and the expected sizes are denoted in the schematic (left). Expected sizes for PfGRP170‐GFP‐DDD clones (blue), parental DNA (red), and plasmid (grey) were observed (right). Parental and plasmid bands were absent from the PfGRP170‐GFP‐DDD clonal cell lines. (f) Western blot analysis of protein lysates from PM1KO (parental) and PfGRP170‐GFP‐DDD clonal cell lines 1B2 and 1B11 is shown. Lysates were probed with anti‐GFP to visualise PfGRP170 and anti‐PfEF1α as a loading control. (g) Asynchronous PfGRP170‐GFP‐DDD parasites were paraformaldehyde fixed and stained with anti‐GFP, anti‐PfGRP78 (BiP), and DAPI to visualise the nucleus. Images were taken as a Z‐stack using super resolution microscopy and SIM processing was performed on the Z‐stacks. Images are displayed as a maximum intensity projection. The scale bar is 2 μm

A blast search to identify ER localised Hsp70 proteins in P. falciparum revealed two proteins, HSP70‐2 (PfGRP78/BiP) and a putative HSP110 (PF3D7_1344200). HSP110 proteins are considered large HSP70 chaperones, having sequence homology to both the nucleotide and substrate‐binding domains of other HSP70 members (Easton, Kaneko, & Subjeck, 2000). The increased size of HSP110 family members is the result of an extended α‐helical domain at the C‐terminus as well as an unstructured loop inserted in the substrate‐binding domain (Behnke & Hendershot, 2014; Easton et al., 2000; Figure 1a). In other eukaryotic organisms, the ER localised HSP110 (referred to as GRP170) is a chaperone with four primary protein domains: a signal peptide, a nucleotide binding domain, a substrate‐binding domain, and an extended C‐terminus (Andreasson et al., 2010; Easton et al., 2000). A protein sequence alignment using the yeast GRP170 (Lhs1) was used to predict the boundaries of these domains in PF3D7_1344200 (PfGRP170; Figure 1a; Figure S1). Most of the sequence conservation between Lhs1 and PfGRP170 was found to be in the nucleotide binding domain (Figure S1). PfGRP170 is well conserved across multiple *Plasmodium* species, including other human malaria‐causing species (Figure S2). This level of conservation decreases in another apicomplexan (*Toxoplasma gondii*) and even more so in yeast and humans (Figure S2).

### 2.2 Generating PfGRP170‐GFP‐DDD conditional mutants

Conditional mutants for PfGRP170 (termed PfGRP170‐GFP‐DDD) were generated by tagging the endogenous PfGRP170 locus at the 3′ end, using single homologous crossover, with a GFP reporter, the E *scherichia* coli DHFR destabilisation domain (DDD), and an ER retention signal (SDEL; Figure 1b). The endogenous PfGRP170 gene encodes a C‐terminus SDEL sequence, a potential ER retention signal, and therefore, we added an SDEL sequence after the DDD domain in order to avoid mislocalisation of the tagged protein. In the presence of the small ligand trimethoprim (TMP), the DDD is maintained in a folded state. However, if TMP is removed from the culture medium, the DDD unfolds and becomes unstable (Beck et al., 2014; Cobb et al., 2017; Florentin et al., 2017; Muralidharan et al., 2012; Muralidharan, Oksman, Iwamoto, Wandless, & Goldberg, 2011). Intramolecular binding of the chaperone to the unfolded domain inhibits normal chaperone function (Figure 1b; Beck et al., 2014; Florentin et al., 2017; Muralidharan et al., 2012). Two independent transfections were carried out, and integrated parasites were selected via several rounds of drug cycling. Polymerase chain reaction (PCR) integration tests following drug selection indicated that the percentage of integrated parasites in both transfections were extremely low (Figure 1c). Consequently, standard limiting dilution could not be used to clone out integrated parasites. To circumvent this issue, flow cytometry was used to enrich and sort extremely rare GFP positive parasites. Despite low enrichments and sorting rates (GFP positive population = ~1.13E‐3), we successfully obtained two clones, termed 1B2 and 1B11, using flow sorting (Figure 1c,d). Proper integration into the *pfgrp170* locus was confirmed by a southern blot analysis (Figure 1e). Western blot analysis revealed that the PfGRP170‐GFP‐DDD protein was expressed at the expected size (Figure 1f). Immunofluorescence assays (IFAs) and western blot analysis showed that the PfGRP170‐GFP‐DDD fusion protein was expressed and localised to the parasite ER during all stages of the asexual life cycle (Figure 1g; Figure S3).

### 2.3 PfGRP170 is essential for asexual growth and surviving febrile episodes

**Figure 2:**
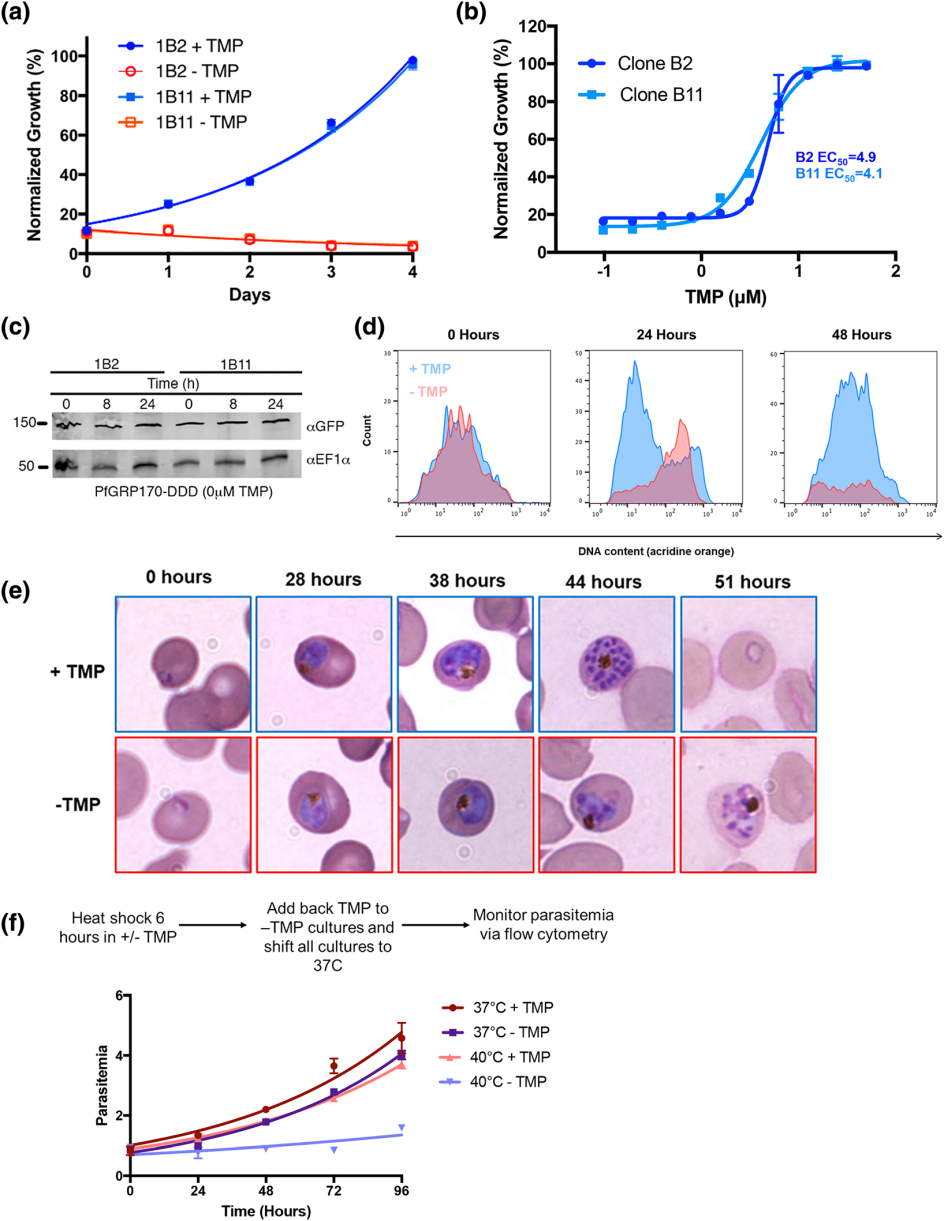
PfGRP170 is essential and required for surviving a heat shock. (a) Growth of asynchronous PfGRP170‐GFP‐DDD clonal cell lines 1B2 and 1B11, in the presence or absence of 20 μM TMP, was observed using flow cytometry over 4 days. One hundred percent growth is defined as the highest parasitemia in samples with TMP, on the final day of the experiment. Data were fit to an exponential growth curve equation. Each data point is representative of the mean of three replicates ± SEM. (b) Asynchronous PfGRP170‐GFP‐DDD clonal cell lines 1B2 and 1B11 were grown in a range of TMP concentrations for 48 hr. After 48 hr, parasitemia was observed using flow cytometry. One hundred percent growth is defined as the highest parasitemia in the presence of TMP on the final day of the experiment. Data were fit to a dose–response equation. Each data point is representative of the mean of three replicates ± SEM. (c) Western blot analysis of PfGRP170‐GFP‐DDD lysates at 0, 8, and 24 hr following the removal of TMP is shown. Lysates were probed with anti‐GFP to visualise PfGRP170 and anti‐PfEF1α as a loading control. (d) Flow cytometric analysis of asynchronous PfGRP170‐GFP‐DDD parasites, incubated with (blue) and without TMP (red), and stained with acridine orange. Data at 0, 24, and 48 hr after the removal of TMP are shown. (e) TMP was removed from tightly synchronised PfGRP170‐GFP‐DDD ring stage parasites and their growth and development through the life cycle was monitored by Hema 3 stained thin blood smears. Representative images are shown from the parasite culture at the designated times. (f) PfGRP170‐GFP‐DDD clones 1B2 and 1B11 were incubated with and without TMP for 6 hr at either 37°C or 40°C. Following the incubation, TMP was added back to all cultures and parasites were shifted back to 37°C. Parasitemia was then observed over 96 hr via flow cytometry. Data were fit to an exponential growth curve equation. Each data point shows the mean of three replicates ± SEM

To investigate the essentiality of PfGRP170, PfGRP170‐GFP‐DDD asynchronous parasites were cultured in the absence of TMP, and parasitemia was observed using flow cytometry. A growth defect was seen within 24 hr after the removal of TMP, resulting in parasite death (Figure 2a). Furthermore, the two clonal parasite lines exhibited a dose‐dependent growth response to TMP (Figure 2b). Consistent with data from other chaperones tagged with the DDD (Beck et al., 2014; Florentin et al., 2017; Muralidharan et al., 2012), TMP removal did not result in degradation of PfGRP170‐GFP‐DDD (Figure 2c). Conditional inhibition of *Plasmodium* proteins that does not involve their degradation has also been observed for other nonchaperone proteins (Ganter et al., 2017; Ito, Schureck, & Desai, 2017). Moreover, the removal of TMP did not affect the ER localisation of PfGRP170 (Figure S4).

Using a nucleic acid stain, acridine orange, we used flow cytometry to specifically observe each stage of the asexual life cycle (ring, trophozoite, and schizont) in PfGRP170‐GFP‐DDD parasites incubated with and without TMP (Figure 2d). The amounts of RNA and DNA increase over the asexual life cycle as the parasite transitions from a ring to trophozoite to a multinucleated schizont. We observed that upon TMP removal, mutant parasites arrested in a relatively late developmental stage (Figure 2d). To identify the stage in the asexual life cycle where the mutant parasites died, TMP was removed from tightly synchronised ring stage cultures and parasite growth, and morphology was assessed over the 48‐hr life cycle. We observed morphologically abnormal parasites late in the lifecycle, when control parasites had undergone schizogony (Figure 2e). The PfGRP170‐GFP‐DDD parasites grown without TMP ultimately failed to progress through schizogony and did not reinvade new RBCs (Figure 2e).

The cytoplasmic ortholog of PfGRP170, PfHSP110, was previously shown to be essential for surviving heat stress (Muralidharan et al., 2012). Therefore, we tested whether PfGRP170 mutants were sensitive to a brief heat shock. Asynchronous parasites were incubated in the absence of TMP for 6 hr at either 37°C or 40°C. Following the 6‐hr incubation, TMP was added back to all cultures, which were then grown at 37°C for two growth cycles, whilst measuring parasitemia every 24 hr. The growth of parasites at 37°C was not significantly affected by the brief removal of TMP (Figure 2f). In contrast, incubating parasites at 40°C without TMP resulted in reduced parasite viability compared with parasites grown at 40°C with TMP (Figure 2f).

### 2.4 PfGRP170 is not required for trafficking of apicoplast proteins

Protein trafficking to the apicoplast is essential for parasite survival. Proteins targeted to the apicoplast contain an N‐terminal transit peptide that is revealed upon signal peptide cleavage in the ER (Tonkin et al., 2008; Waller, Reed, Cowman, & McFadden, 2000). It remains unclear whether apicoplast‐targeted proteins go through the Golgi before reaching their final destination. It has been shown that disruption of ER to Golgi trafficking, using brefeldin A (BFA), does not reduce apicoplast transport (Tonkin et al., 2008; Tonkin, Struck, Mullin, Stimmler, & McFadden, 2006). One of these studies further demonstrated that the addition of an ER retention sequence (SDEL), to a GFP with a transit peptide, did not reduce apicoplast trafficking or transit peptide cleavage (Tonkin et al., 2006). However, a separate but similar analysis came to the opposite conclusion (Heiny, Pautz, Recker, & Przyborski, 2014). Thus, the identification, packaging, and transport of apicoplast‐targeted proteins from the ER remain unanswered questions.

**Figure 3:**
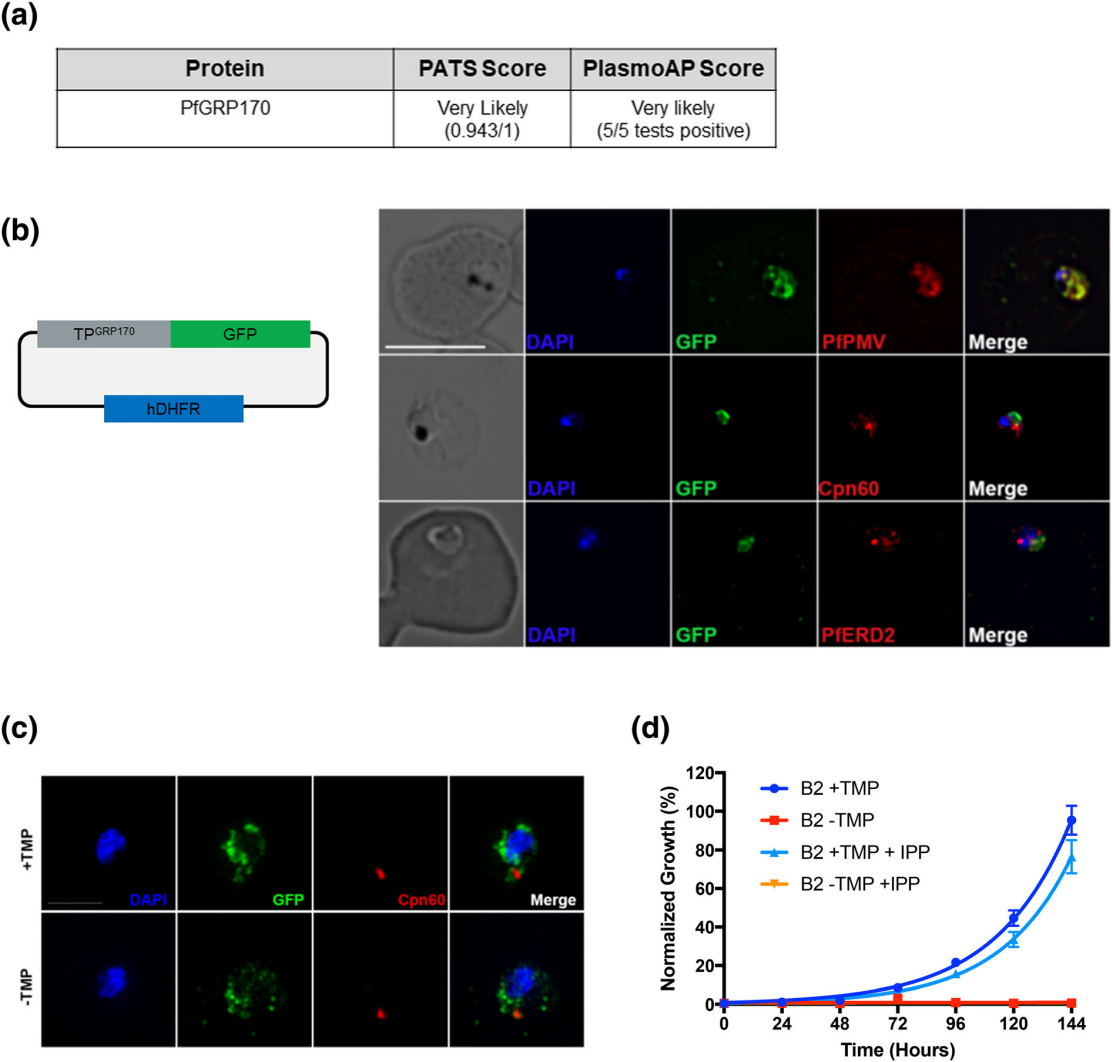
Putative PfGRP170 apicoplast transit peptide localises to the ER and conditional inhibition of PfGRP170 does not affect trafficking of apicoplast proteins. (a) Analysis of PfGRP170's protein sequence using two apicoplast transit peptide prediction programmes: prediction of apicoplast‐targeted sequences (PATS) and PlasmoAP. (b) PfGRP170's putative apicoplast transit peptide was fused to GFP and transfected into 3D7 parasites. Parasites were fixed with acetone and stained with DAPI, anti‐GFP (to label the PfGRP170 putative transit peptide) and either anti‐PfPMV (ER), anti‐PfERD2 (Golgi), or anti‐Cpn60 (apicoplast) to determine subcellular localisation. The images were taken with Delta Vision II, deconvolved, and are displayed as a maximum intensity projection. The scale bar is 5 μm. (c) Synchronised ring stage PfGRP170 parasites were incubated for 24 hr with and without TMP. Following the incubation, the parasites were fixed with paraformaldehyde and stained with DAPI, anti‐GFP (PfGRP170), and anti‐Cpn60 (apicoplast). Images were taken as a Z‐stack using super resolution microscopy and SIM processing was performed on the Z‐stacks. Images are displayed as a maximum intensity projection. The scale bar is 2 μm. (d) Asynchronous PfGRP170‐GFP‐DDD parasites were incubated with and without TMP and in the presence or absence of 200 μM IPP. Parasitemia was monitored using flow cytometry for 144 hr. One hundred percent growth is defined as the highest parasitemia in the presence of TMP, on the final day of the experiment. Data were fit to an exponential growth curve equation. Each data point is representative of the mean of three replicates ± SEM

Despite our observation of a definite ER localisation, two software analysis tools (prediction of apicoplast‐targeted sequences and PlasmoAP) predicted a strong apicoplast transit peptide for PfGRP170 (Figure 1g; Figure 3a). We were therefore interested to find out whether PfGRP170 plays a role in apicoplast trafficking. We tested whether the putative PfGRP170 transit peptide (amino acids: 1 to 150) could be trafficked to the apicoplast by episomally expressing the predicted PfGRP170‐transit peptide fused to a GFP reporter without an ER retention signal (Figure 3b). We performed colocalisation assays using ER, apicoplast, and Golgi markers and determined that the putative transit peptide localised to the ER due to colocalisation with ER marker plasmepsin V (Figure 3b).

To determine the role of PfGRP170 in trafficking proteins to the apicoplast, we removed TMP from PfGRP170‐GFP‐DDD parasites and examined the localisation of the apicoplast‐localised cpn60 (Fellows, Cipriano, Agrawal, & Striepen, 2017; Florentin et al., 2017; Sheiner et al., 2011). No defects in apicoplast localisation of cpn60 were observed (Figure 3c). Additionally, incubation with the essential apicoplast metabolite IPP (Yeh & DeRisi, 2011) failed to rescue or have any positive effect on TMP removal in PfGRP170‐GFP‐DDD parasites (Figure 3d).

### 2.5 Interactions of PfGRP170

**Figure 4:**
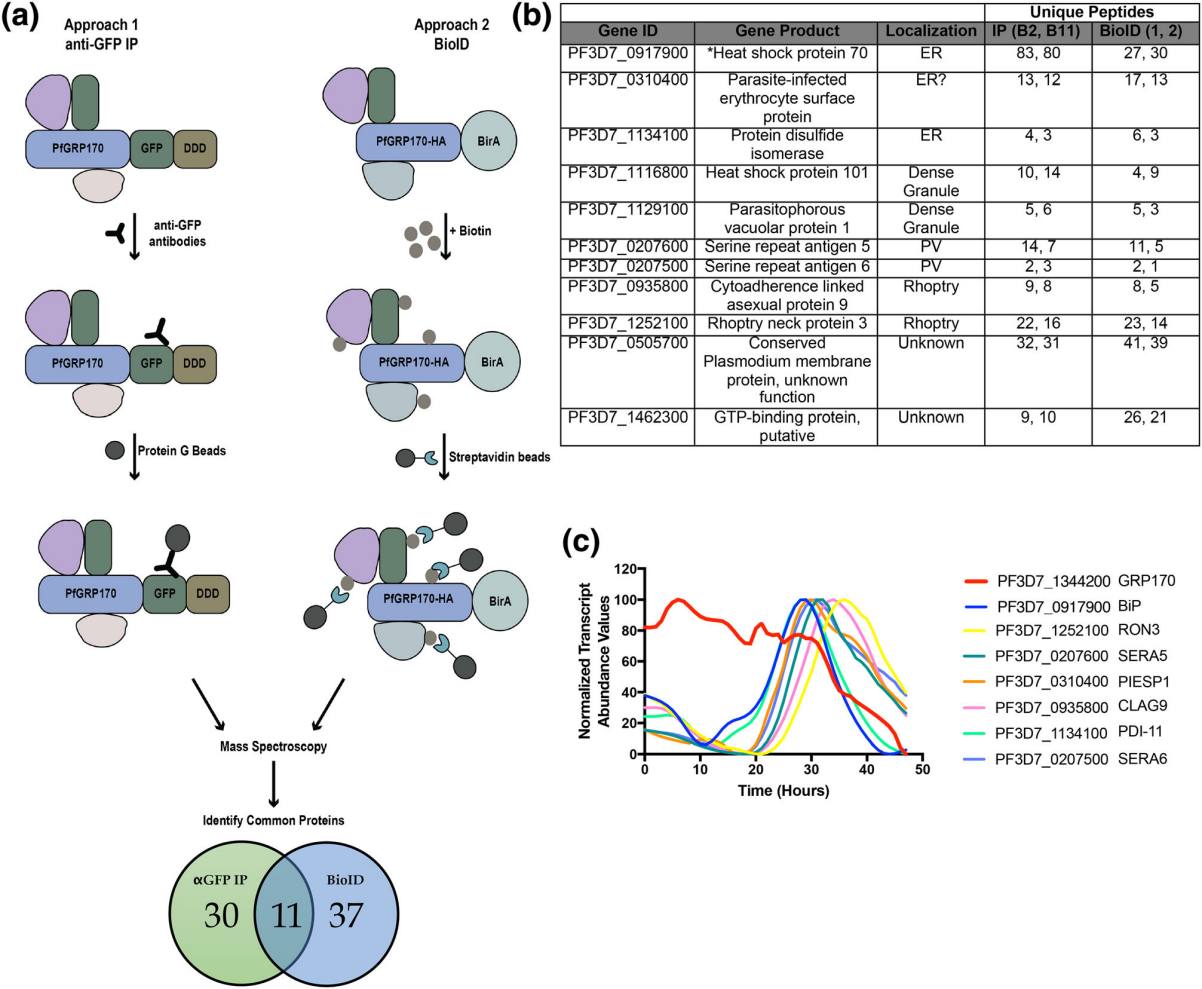
PfGRP170 interacting partners. (a) Schematic diagram illustrating the two independent methods used to identify potential interacting partners of PfGRP170: anti‐GFP IP using lysates from PfGRP170‐GFP‐DDD parasites and streptavidin IP of PfGRP170‐BirA parasites incubated with biotin for 24 hr followed by mass spectroscopy. The proteins identified from each IP were filtered to include only proteins that had a signal peptide and/or transmembrane domain using PlasmoDB. Proteins found in the respective control IP's (excluding PfBiP) were also removed from further analysis (data in Table S1). (b) The 11 proteins identified in both independent mass spectroscopy approaches (see Figure 4a; Table S1). The PlasmoDB gene ID, gene product, putative subcellular localisation, and number of unique peptides identified for each protein in each independent experiment are listed. (c) The relative transcript abundance of interacting proteins, with peak expression around the time the PfGRP170‐GFP‐DDD parasites die (36–44 hr), are plotted using genome‐wide real‐time transcript data (Painter et al., 2018)

Two independent approaches were taken to identify the interacting proteins of PfGRP170 (Figure 4a). The first was an anti‐GFP immunoprecipitation (IP) followed by mass spectroscopy. In the second approach, we generated a parasite line episomally expressing PfGRP170 tagged with an HA, the promiscuous biotin ligase (BirA), and an ER retention signal (KDEL). When exogenous biotin is added to the PfGRP170‐HA‐BirA parasites, the BirA tagged protein will biotinylate interacting proteins or those that are in close proximity (Chen et al., 2015). These biotinylated proteins were isolated using streptavidin coated magnetic beads. A western blot analysis confirmed expression of the PfGRP170‐HA‐BirA fusion protein (Figure S5a). Colocalisation IFA's confirmed that the PfGRP170‐HA‐BirA protein localises to the parasite ER (Figure S5b). Additionally, western blot analysis demonstrates that proteins are biotinylated in the PfGRP170‐HA‐BirA parasite lines when biotin is added (Figure S5c).

Mass spectroscopy was used to identify PfGRP170 interacting proteins from two independent anti‐GFP IP's of PfGRP170‐GFP‐DDD parasites and two biological replicates of streptavidin pulldowns from PfGRP170‐HA‐BirA parasites following an incubation with biotin (Table S1). Further, two independent anti‐GFP IP's from the PM1 parental control and one streptavidin pull down from 3D7 parasites incubated with biotin to filter out non‐specific interactions (Table S1). Both BiP and PfGRP170 were found in the control IP's, albeit in lower peptide counts (Table S1). This is not surprising as chaperones are common contaminants in mass spectroscopy. However, due to the documentation of PfGRP170 being an interactor and regulator of BiP function, we opted to keep BiP in our analysis (Andreasson et al., 2010). In order to obtain a list of proteins specific to the ER and parasite secretory pathway, proteins identified by mass spectroscopy in each IP were filtered to include only those, which had a signal peptide or transmembrane domain. Thirty proteins were found in both the 1B2 and 1B11 in the anti‐GFP IP's, and 37 proteins were found in the two replicates of the PfGRP170‐HA‐BirA streptavidin pull down (Figure 4a). Of these, 11 proteins were identified using both approaches suggesting that these are true interactors of PfGRP170 (Figure 4b). Using recently published real‐time transcriptional abundance data, we plotted the normalised transcriptional abundance values for all 11 proteins (Painter et al., 2018; Figure S6). Upon removal TMP, the PfGRP170‐DDD parasites die 38–44 hr postinvasion (Figure 2e), and therefore, it is likely that the essential function of PfGRP170 is linked to proteins expressed during these late stages of the asexual life cycle. Excluding proteins that were expressed earlier in the life cycle narrow the list of putative essential interactors of PfGRP170 to the seven proteins (Figure 4c; Painter et al., 2018).

### 2.6 PfGRP170 is not required for trafficking to the RBC

In order for the parasite to grow, develop, and divide, it must drastically remodel the host RBC (Boddey & Cowman, 2013). These modifications are accomplished through the export of proteins from the ER to the RBC. In model eukaryotes, such as yeast and mammalian cells, molecular chaperones, and specifically those that are ER‐localised, play central roles in protein trafficking (Araki & Nagata, 2011; Gidalevitz et al., 2013). Therefore, we tested whether conditional inhibition of PfGRP170 would prevent trafficking of several exported proteins (PfHSP70x, PfMAHRP1, and FIKK4.2). Our results demonstrate that loss of PfGRP170 function did not affect the localisation of these proteins to the host RBC (Figure S7a–c).

### 2.7 PfGRP170 and BiP interact

**Figure 5:**
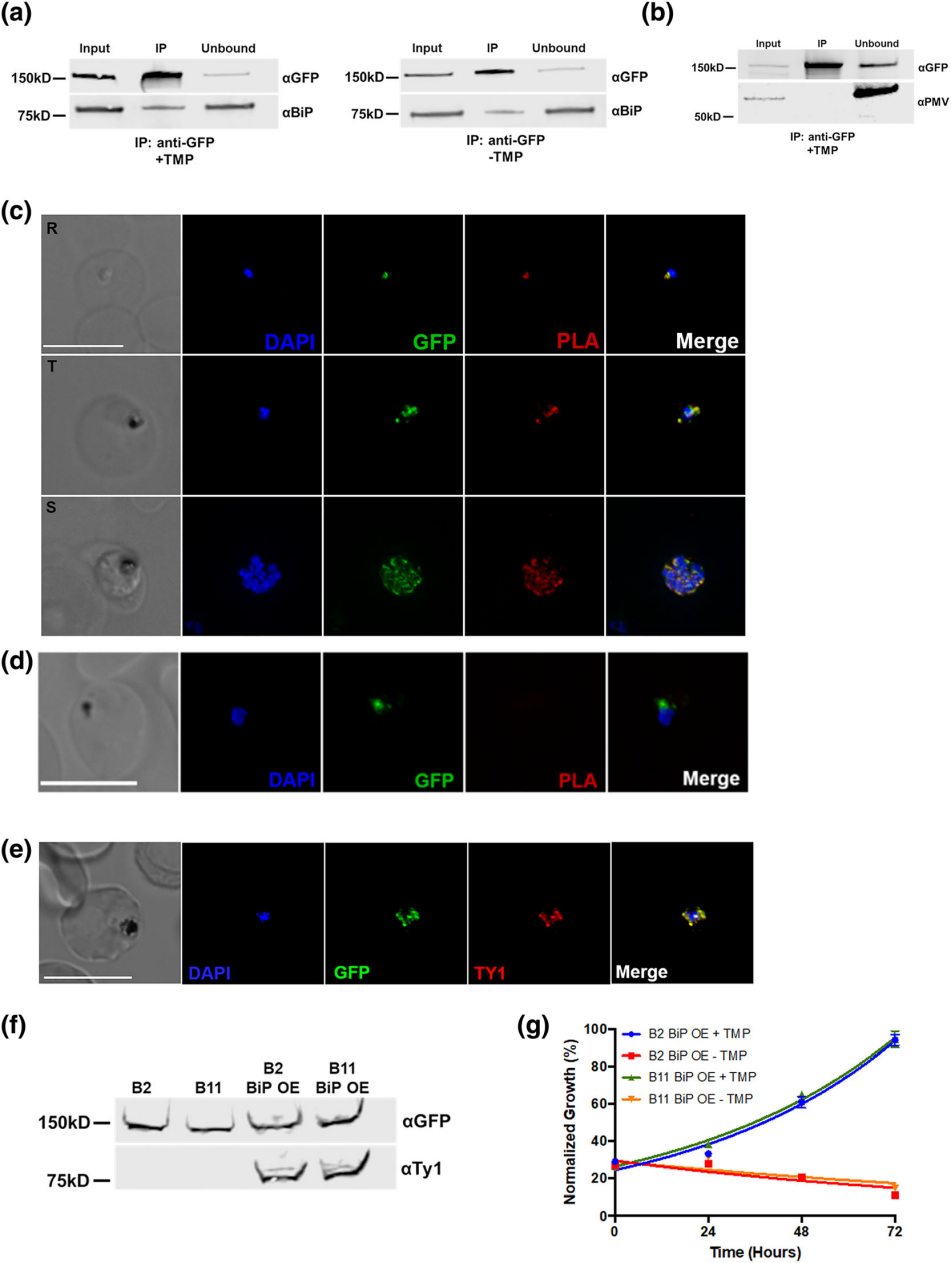
PfGRP170 Interacts with BiP. (a) Synchronised ring stage PfGRP170‐GFP‐DDD parasites were incubated with and without TMP for 24 hr. Following this incubation, an anti‐GFP IP was performed, and input, IP, and unbound fractions were analysed using a western blot. The blot was probed using anti‐GFP and anti‐BiP. (b) Western blot analysis of an anti‐GFP IP performed on asynchronous PfGRP170‐GFP‐DDD parasites. Input, IP, and unbound fractions are shown. The blot was probed using anti‐GFP and anti‐PfPMV. (c) In vivo interaction of PfGRP170 and BiP. PfGRP170‐GFP‐DDD parasites were paraformaldehyde fixed and stained with anti‐GFP and anti‐BiP. A proximity ligation assay (PLA) was then performed. The scale bar is 5 μm. A negative control using anti‐GFP and anti‐PfPMV is shown in (d). (e) Asynchronous PfGRP170‐GFP‐DDD parasites overexpressing PfBiP‐Ty1 were paraformaldehyde fixed and stained with anti‐GFP (PfGRP170), anti‐Ty1 (PfBiP‐Ty1‐KDEL), and DAPI to visualise the nucleus. The images were taken with Delta Vision II, deconvolved, and are displayed as a maximum intensity projection. The scale bar is 5 μm. (f) Western blot analysis of protein lysates from parental 1B2 and 1B11 parasites as well as 1B2 and 1B11 parasites overexpressing the PfBiP‐Ty1fusion protein. Lysates were probed with anti‐GFP to visualise PfGRP170 and anti‐Ty1 to visualise PfBiP‐Ty1‐KDEL. (g) Parasitemia of asynchronous PfGRP170‐GFP‐DDD parasites expressing PfBiP‐Ty1‐KDEL, in the presence or absence of 20 μM TMP, was observed using flow cytometry over 3 days. One hundred percent growth is defined as the highest parasitemia on the final day of the experiment. Data were fit to an exponential growth curve equation. Each data point is representative of the mean of three replicates ± SEM

One of the most abundant proteins identified in our mass spectroscopy data was PfBiP (Figure 4b; Table S1). However, PfBiP was also found in our IPs performed with parental controls; therefore, we were tested whether PfGRP170 and PfBiP interact in P. falciparum. We performed an anti‐GFP co‐IP and probed the lysate for PfBiP. We observed that PfGRP170 and PfBiP interact and this interaction is not lost upon TMP removal (Figure 5a). As a control, we probed the GFP co‐IP lysates for a different ER protein, plasmepsin V (PMV), and found that it did not pull down with PfGRP170 (Figure 5b).

To visualise the PfGRP170‐PfBiP interaction within the cellular context of the infected RBC, we utilised a proximity ligation assay (PLA; Fredriksson et al., 2002; Gullberg et al., 2004; Söderberg et al., 2006). The PLA positive signal indicates that two proteins are within 40 nm of each other, suggesting a close interaction within the cell. This approach has been used successfully in *Plasmodium* to demonstrate interaction of exported proteins (Külzer et al., 2012). We performed this assay using anti‐GFP and anti‐BiP antibodies and observed a positive signal at all life cycle stages (Figure 5c). As a negative control, we also probed with an antibody against the ER localised protease PMV, and despite the colocalisation of these two proteins in the ER, we did not see a positive PLA signal, suggesting distinct suborganellar localisations (Figure 5d). Together, these results demonstrate that PfGRP170 and PfBiP interact during all stages of the asexual life cycle of P. falciparum.

The function of BiP is critical for ER biology and in other eukaryotes its function is regulated by GRP170 (Andreasson et al., 2010; de Keyzer et al., 2009). Additionally, loss of the PfGRP170 yeast homologue, Lhs1, activates a stress response mechanism known to upregulate BiP expression (Tyson & Stirling, 2000). Therefore, we tested whether the PfGRP170‐GFP‐DDD mutants could be rescued by overexpression of PfBiP. We did this by episomally expressing PfBiP with a Ty1 tag and an ER retention signal (KDEL) in the PfGRP170‐GFP‐DDD mutants. Colocalisation assays demonstrate that the PfBiP‐Ty1 fusion protein is targeted to the ER, and we observe that the protein is expressed at the expected size by western blot (Figure 5e,f). To determine if the overexpression of PfBiP could rescue parasite growth during TMP removal, the PfGRP170‐GFP‐DDD parasites expressing the PfBiP‐Ty1 protein were incubated with and without TMP, and the parasitemia was monitored using flow cytometry. We demonstrate that the overexpression of PfBiP in the PfGRP170‐GFP‐DDD parasites could not rescue parasite growth (Figure 5g).

### 2.8 Loss of PfGRP170 function activates the PK4 stress response pathway

**Figure 6:**
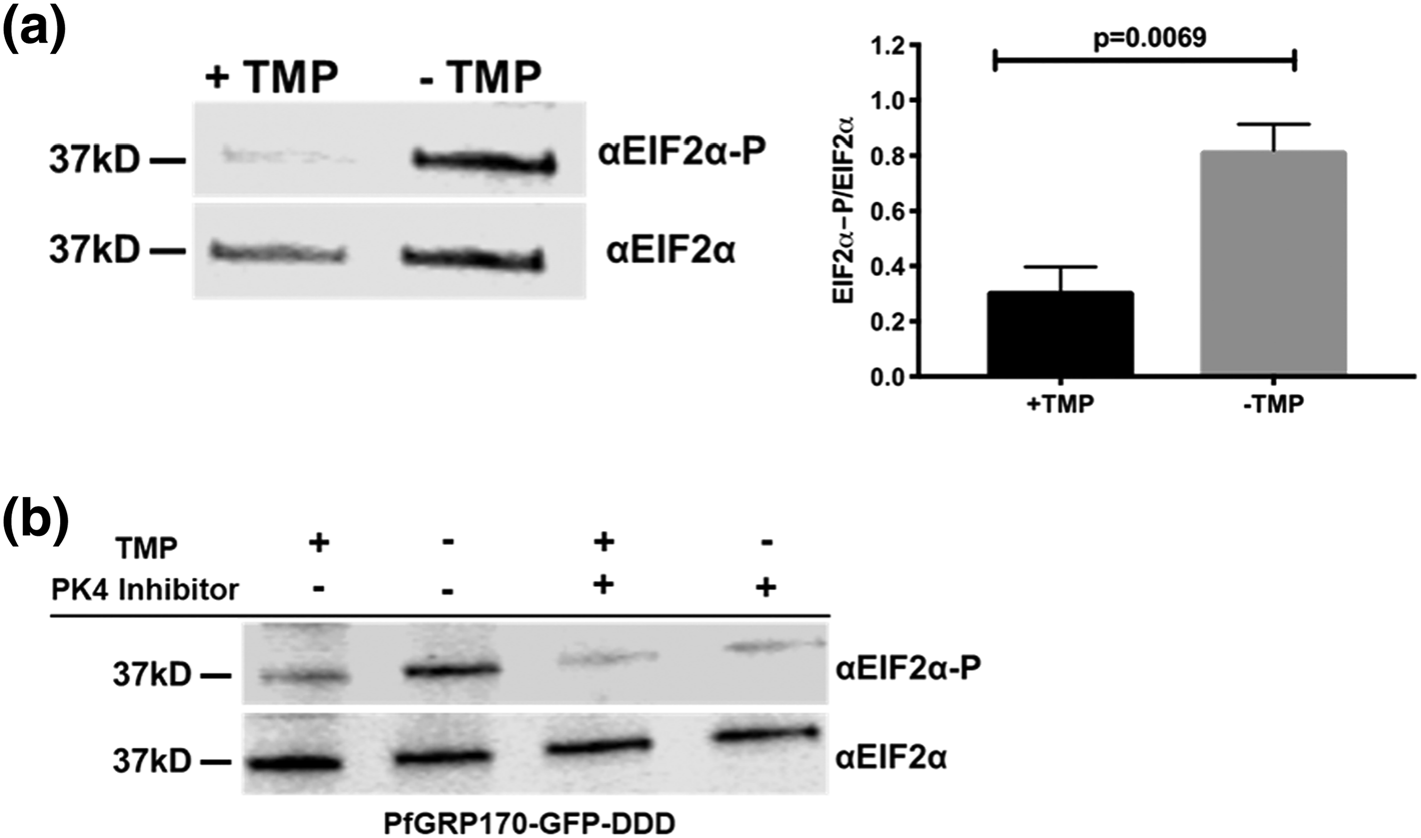
Loss of PfGRP170 function activates the PK4 stress pathway. (a) (Left) Synchronised ring stage PfGRP170‐GFP‐DDD parasites were incubated with and without TMP for 24 hr. Protein was isolated from these samples and analysed via western blot, probing for anti‐eIF2α and anti‐Phospho‐eIF2α. (Right) The ratio of phosphorylated EIF2α over total EIF2α for PfGRP170‐GFP‐DDD parasites incubated with and without TMP is shown. Western blot band intensities were calculated using ImageJ software (NIH), and the significance was calculated using an unpaired t test. Data are representative of four biological replicates ± SEM. (b) Synchronised ring stage PfGRP170‐GFP‐DDD parasites were incubated with and without TMP and in the presence and absence of 2 μM PK4 inhibitor GSK2606414 for 24 hr. Protein was isolated from these samples and analysed via western blot by probing for anti‐eIF2a and anti‐Phospho‐eIF2α

In addition to their function in the secretory pathway, molecular chaperones perform a vital role in the management of cellular stress. *Plasmodium* lack much of the ER machinery used to activate stress response pathways (Chaubey, Grover, & Tatu, 2014; Harbut et al., 2012; Zhang et al., 2012). The only identified ER stress response pathway in *Plasmodium* is the PERK/PK4 pathway (Zhang et al., 2012; Zhang et al., 2017). Signalling through this pathway has been shown to occur in the parasite following artemisinin treatment (Zhang et al., 2017). Under normal conditions, PK4 exists as a transmembrane monomeric protein in the ER. When the ER is stressed, PK4 oligomerises and becomes active, phosphorylating the cytoplasmic translation initiation factor EIF2‐α to halt translation and flux through the ER (Zhang et al., 2012; Zhang et al., 2017). To determine whether this pathway was activated during conditional inhibition of PfGRP170, PfGRP170‐GFP‐DDD parasites were tightly synchronised to the ring stage and grown without TMP for 24 hr, after which parasite lysate was collected and the phosphorylation state of EIF2‐α determined by western blot. We observed that PfGRP170 auto‐inhibition resulted in the phosphorylation of EIF2‐α, indicating that this pathway was activated (Figure 6a).

Because conditional inhibition of PfGRP170 resulted in EIF2‐α phosphorylation, which has been shown to be required for resistance to artemisinin resistance, we tested if PfGRP170 plays a role in drug resistance. For this purpose, we utilised PfGRP170‐BirA parasites, which have an extra copy of PfGRP170. Using the ring‐stage survival assay, we compared the growth of the parental parasite line (3D7) with that of PfGRP170‐BirA parasites after brief exposure to artemisinin. Our data show that overexpression of PfGRP170 did not result in artemisinin resistance (Figure S8a,b).

Several *Plasmodium* kinases have been shown to phosphorylate EIF2‐α in late developmental stages or in response to other cellular stress or artemisinin treatment (Babbitt et al., 2012; Fennell et al., 2009; Ward, Equinet, Packer, & Doerig, 2004; Zhang et al., 2012). We were therefore interested in identifying the specific kinase that is responsible for the phosphorylation of EIF2‐α during conditional inhibition of PfGRP170. The ER kinase, PK4, has been shown to be activated by ER stress in *Plasmodiu*m (Chaubey et al., 2014). Therefore, we incubated synchronised PfGRP170‐GFP‐DDD parasites without TMP for 24 hr, in the presence or absence of a specific PK4 inhibitor GSK2606414 (Zhang et al., 2017). Parasite lysates were used to determine the phosphorylation state of EIF2‐α. We observed that in the presence of the PK4 inhibitor, EIF2‐α phosphorylation was blocked, demonstrating that conditional inhibition of PfGRP170 specifically results in PK4 activation, which leads to phosphorylation of EF2‐α (Figure 6b). As a control, we used the parental strain, PM1, and incubated these parasites with and without TMP or the PK4 inhibitor (Figure S9). This experiment showed no changes in levels of EIF2‐α regardless of the presence of TMP or the PK4 inhibitor.

## 3 DISCUSSION

We present in this work the first characterisation of PfGRP170 in the asexual life cycle of P. falciparum. We have generated conditional mutants that allow us to probe the role of this protein using the DDD conditional auto‐inhibition system (Cobb et al., 2017; Florentin et al., 2017; Ganter et al., 2017; Ito et al., 2017; Muralidharan et al., 2011; Muralidharan et al., 2012). Additionally, taking advantage of the GFP fused to PfGRP170, we were able to isolate an exceptionally rare clonal population using flow cytometry. This technique achieved what a traditional limiting dilution method could not. Moreover, this type of flow sorting can be implemented not only for rare events but also to significantly cut down the time from transfection to a clonal cell population.

We demonstrate here that PfGRP170 is an ER resident protein that is essential for asexual growth in P. falciparum. Loss of PfGRP170 function leads to a growth arrest of parasites late in development and their subsequent death. In yeast and mammals, GRP170 functions in a complex with the ER chaperone BiP, serving as the nucleotide exchange factor to regulate BiP activity (Andreasson et al., 2010; de Keyzer et al., 2009). Unlike P. falciparum, Yeast null for GRP170 are viable due to the upregulation of Sil1, another nucleotide exchange factor, that usually plays a role in the IRE1 stress response pathway (Tyson & Stirling, 2000). The *Plasmodium* genome does not encode Sil1 and IRE1, which aligns with the observed essentiality of PfGRP170 during the blood stages. Additionally, research in mammalian systems suggests that GRP170 also has BiP‐independent functions, such as binding unfolded substrates (Behnke & Hendershot, 2014). Our data show, via IP, mass spectroscopy, and PLAs, that PfGRP170 interacts with BiP in P. falciparum suggesting that it regulates BiP function. Further, overexpression of PfBiP was unable to rescue loss of PfGRP170 function and the conditional inhibition of PfGRP170 does not reduce its interaction with PfBiP. These data suggest that a PfBiP independent function of PfGRP170 is essential for parasite survival.

Previously, it was shown that apicoplast transit peptides are predicted to bind the ER chaperone BiP, and when these predicted binding sites were mutated, targeting to the apicoplast was disrupted (Foth et al., 2003). Moreover, an Hsp70 inhibitor with an antimalarial activity was shown to inhibit apicoplast targeting (Ramya, Karmodiya, Surolia, & Surolia, 2007; Ramya, Surolia, & Surolia, 2006). These data, combined with the predicted transit peptide of PfGRP170, led us to investigate the role of this chaperone in apicoplast trafficking. Interestingly, when the putative transit peptide was tagged with a GFP reporter and without an ER retention signal, the fusion protein was retained in the ER. It was previously reported that proteins with a signal peptide and no ER retention signal are secreted to the parasitophorous vacuole (Boddey et al., 2016; Gruring et al., 2012; Hiller et al., 2004; Marti, Good, Rug, Knuepfer, & Cowman, 2004). However, it was also shown that some proteins with a signal peptide and GFP (lacking an ER retention or trafficking signals) remain in the parasite ER (Boddey et al., 2016). Regardless, this reporter was not sent to the apicoplast indicating that it is not a functional apicoplast transit peptide. Previous work suggest that appending the first 137 amino acids of PfGRP170 to a GFP reporter (without a retention signal) resulted in this chimeric protein localising partially to the apicoplast and to the parasitophorous vacuole (Heiny, Spork, & Przyborski, 2012). Our chimeric protein includes the first 150 amino acids of PfGRP170, which may account for some of the differences in the two studies. In addition, PfGRP170 auto‐inhibition did not lead to any defects in trafficking to the apicoplast, nor could it be rescued with the essential apicoplast metabolite IPP. Further, we did not identify any apicoplast‐localised proteins as potential interactors of PfGRP170. These data suggest that the primary function of PfGRP170 does not function in the apicoplast trafficking pathway.

Protein trafficking to the host RBC originates in the parasite ER and is essential for parasite viability and therefore could potentially account for the observed death phenotype during conditional inhibition of PfGRP170 (Boddey & Cowman, 2013; Deponte et al., 2012). PfGRP170 was shown to associate with exported proteins in another study that identified proteins that bind to the antigenic variant surface protein, PfEMP1 (Batinovic et al., 2017). However, our data show that there is no significant difference in the trafficking of some exported proteins upon conditional inhibition of PfGRP170, suggesting that protein export is not blocked.

ER chaperones are known in other eukaryotes to be vital to managing cellular stress (Walter & David, 2011; Xu et al., 2005). However, several ER localised stress response pathways present in other eukaryotes are absent in P. falciparum, and few molecular players in the parasite ER stress response pathway are known. Our data demonstrate that PfGRP170 is important for coping with a specific form of cellular stress, namely, heat shock. This finding highlights a potential critical role for PfGRP170 in vivo, as high febrile episodes are one of the main symptoms of clinical malaria and are considered a defence mechanism against parasites. GRP170 in mammalian systems has been shown to bind to the transmembrane proteins in the ER that are involved in the unfolded protein response (UPR), suggesting it may regulate these pathways (Sanson et al., 2009; Wang et al., 2011). The *Plasmodium* genome does not encode many of the UPR orthologues, but a single ER stress pathway (PK4 signalling) has been previously described and was shown to be activated following artemisinin treatment (Zhang et al., 2012; Zhang et al., 2017). Here, we demonstrate that loss of PfGRP170 function results in the activation of PK4 stress pathway, providing the first link between an endogenous ER resident protein and the activation of the PK4 pathway in P. falciparum. Further, our data suggest that even though the PK4 stress response pathway is activated upon removal of TMP, this pathway is ultimately unable to prevent parasite death. This is most likely because one or more essential proteins that depend of PfGRP170 for their correct folding and function.

Yeast null for the GRP170 homologue, Lhs1, activate the IRE1 UPR signalling pathway (Tyson & Stirling, 2000). Activation of the IRE1 pathway in eukaryotes, which is not present in *Plasmodium*, typically leads to the upregulation of ER chaperones such as BiP (Chaubey et al., 2014; Harbut et al., 2012; Tyson & Stirling, 2000; Yoshida, Matsui, Yamamoto, Okada, & Mori, 2001). These data suggest that the only essential function of Lhs1 is to serve as a nucleotide exchange factor for BiP (Tyson & Stirling, 2000). Therefore, we tested whether the death phenotype seen in the conditional PfGRP170 mutants could be rescued by overexpressing PfBiP. Our experiments revealed that overexpression of PfBiP does not improve viability of the PfGRP170‐GFP‐DDD parasites following TMP removal. These data imply that unlike its homologues in other eukaryotes, the essential function of PfGRP170 may not be entirely linked to its role in regulating BiP.

We utilised two separate IP/mass spectroscopy approaches to generate a list of 11 high‐confidence interacting partners of PfGRP170. Seven of the proteins (including PfBiP) have a peak expression pattern around the time PfGRP170‐GFP‐DDD parasites begin to die. SERA5 and SERA6 have been shown to be required for egress from the RBC, which would be after the PfGRP170‐GFP‐DDD parasites die (Collins, Hackett, Atid, Tan, & Blackman, 2017; Ruecker et al., 2012; Thomas et al., 2018). RON3 has been shown to been suggested to be a protein important for RBC invasion, which implies this protein interaction is also not why PfGRP170‐GFP‐DDD parasites are dying (Zhao et al., 2014). CLAG9, another identified protein, has been proposed to play a role in cytoadherence to CD36 and remodelling the host RBC after invasion by a merozoite (Ling et al., 2004; Trenholme et al., 2000). PDI‐11 was predicted to be non‐essential in a *piggyBac* mutagenesis conducted in *Plasmodium* (Balu, Shoue, Fraser, & Adams, 2005). The remaining protein was parasite‐infected erythrocyte surface protein 1 (PIESP1). Overexpression data suggest that PIESP1 is exported to the host RBC (van Ooij et al., 2008). However, this protein has a putative ER retention signal (TDEL). These last four amino acids were left off of the GFP fusion protein that was expressed in the parasite as the authors predicted this protein was a transmembrane protein and thus leaving off these amino acids would have no effect on protein localisation (van Ooij et al., 2008). Further studies will be needed to determine the precise subcellular localisation of PIESP1 and determine its role in parasite biology. These data show that PfGRP170 is essential for the asexual lifecycle of P. falciparum and that the biological role of PfGRP170 is quite divergent from other eukaryotes. Further, given the divergence between mammalian and parasite GRP170s, PfGRP170 could be a viable antimalarial drug target.

## 4 EXPERIMENTAL PROCEDURES

### 4.1 Primers and plasmid construction

All primer sequences used in this study can be found in Table S2.

Generation of pGDB‐SDEL plasmid was done using the QuikChange II Site‐Directed Mutagenesis Kit (Agilent Technologies) on the pGDB plasmid with primers P1 and P2 per the manufacturer's protocol (Muralidharan et al., 2011).

Genomic DNA was isolated using the QIAamp DNA blood kit (Qiagen). gDNA used in this study was isolated from either 3D7 or plasmepsin I knockout parasites (PM1KO; Muralidharan et al., 2011). The pPfGRP170‐GFP‐DDD plasmid used to generate the PfGRP170‐GFP‐DDD mutants was made by amplifying via PCR an approximately 1‐kb region homologous to the 3′end of the PfGRP170 gene (stop codon not included) using primers P3 and P4. The amplified product was inserted into pGDB‐SDEL plasmid using restriction sites Xho1 and AvrII (New England Biolabs) and transformed into bacteria. The construct was sequenced prior to transfection.

The pGRP170‐HA‐BirA‐KDEL plasmid was prepared by amplifying PfGRP170 (without the stop codon) from 3D7 gDNA using primers P5 and P6 and 3xHA‐BirA from the pTYEOE‐3XHA‐BirA plasmid (from D. Goldberg) using primers P7 and P8. Both PCR products generated included homologous regions used for Sequence and Ligation Independent Cloning (SLIC; Li & Elledge, 2007).The primers to amplify the 3xHA‐BirA included the sequence of an ER retention signal (KDEL). These PCR products were fused together using PCR sewing as described previously and subsequently PCR amplified using primers P5 and P8 (Cobb et al., 2017). The resulting product was then inserted into pCEN‐DHFR (Iwanaga, Kato, Kaneko, & Yuda, 2012) that was digested with Nhe1 and BglII (New England Biolabs) using SLIC and transformed into bacteria as described previously (Cobb et al., 2017; Florentin et al., 2017).

The pPfGRP170TP‐GFP plasmid was prepared by amplifying the first 450 bp (includes the signal peptide and putative transit peptide sequence) of PfGRP170 from PM1 gDNA using primers P5 and P9. The GFP sequence used was amplified from pGDB using primers P10 and P11. The PfGRP170 transit peptide PCR was digested with Nhe1 and AatII (New England Biolabs), and the GFP PCR was digested with AatII and BglII (New England Biolabs). The two fragments were then ligated together (via the AatII digest site) using a T4 ligase (kit from New England Biolabs) and subsequently PCR amplified using primers P5 and P11. The resulting product was then digested with Nhe1 and BglII and inserted into pCEN‐DHFR (Iwanaga et al., 2012) that was digested with Nhe1 and BglII (New England Biolabs) using a T4 ligase and transformed into bacteria as described previously (Cobb et al., 2017; Florentin et al., 2017).

The pPfBiP‐Ty1overexpression plasmid was prepared first by generating cDNA using the SuperScript III reverse transcriptase kit (Invitrogen) using primer P14. PfBiP was then amplified from the cDNA using primers P14 and P15. The resultant PCR product included PfBiP, a single Ty1 tag, and an ER retention signal (KDEL). The pCEN vector was modified to contain the DHOD resistance gene instead of the DHFR for parasite selection (Iwanaga et al., 2012). The PfBiP‐Ty1‐KDEL‐KDEL PCR was cloned into the pCEN‐DHOD vector cut with Nhe1 and BglII (New England Biolabs) using the IN‐Fusion HD EcoDry Cloning Kit (Clontech).

### 4.2 Cell culture, transfections, and isolation of clonal cell lines

Parasites were grown in RPMI 1640 media supplemented with Albumax 1 (Gibco) and transfected as described previously (Beck et al., 2014; Cobb et al., 2017; Florentin et al., 2017; Muralidharan et al., 2011; Muralidharan et al., 2012).

To generate PfGRP170‐GFP‐DDD mutants, PM1KO parasites were transfected with the pPfGRP170‐GFP‐DDD plasmid in duplicate. PM1KO parasites contain the human dihydrofolate reductase (hDHFR) expression cassette, which gives the parasites resistance to TMP (Muralidharan et al., 2011). Drug selection and cycling were performed as described previously using 10 μM TMP (Sigma) and 2.5 μg ml^−1^ blasticidin (Sigma; Florentin et al., 2017; Muralidharan et al., 2011; Muralidharan et al., 2012). Following drug cycling, GFP positive cells were enriched using an S3 Cell Sorter (Bio‐Rad). Individual GFP positive cells from a single transfection were cloned into 96‐well plates using a MoFlo XDP flow cytometer. After the EC_50_ of TMP was determined for clones 1B2 and 1B11, parasites were shifted into media containing 2.5 μg ml^−1^ BSD and 20 μM TMP to facilitate optimal growth.

The PfGRP170‐BirA and PfGRP170TP‐GFP parasites were generated by transfecting 3D7 parasites with plasmids pGRP170‐HA‐BirA‐KDEL or pPfGRP170TP‐GFP, respectively. Parasites expressing these episomal constructs were selected using 2.5 nM WR99210.

To generate the PfGRP170‐GFP‐DDD parasites episomally expressing PfBiP‐Ty1‐KDEL‐KDEL, PfGRP170‐GFP‐DDD clones 1B2 and 1B11 were each transfected with pPfBiP‐Ty1‐KDEL. This plasmid expresses PfBiP‐Ty1‐KDEL using the *pbef1α* bidirectional promoter. Parasites expressing this episomal construct were selected using 250 nM of DSM1 (Ganesan et al., 2011).

### 4.3 Integration tests for PfGRP170‐GFP‐DDD mutants

Genomic DNA was isolated from parasites using the QIAamp DNA blood kit (Qiagen). Control primers to amplify the genome were P4 and P12, and primers used to amplify integrated DNA were P12 and P13.

Southern blot analysis was performed on DNA isolated from PfGRP170‐GFP‐DDD parasites (1B2 and 1B11) as described previously (Cobb et al., 2017; Florentin et al., 2017). The assay was also performed on PM1KO parental DNA and the pGRP170‐DDD plasmid as a control. DNA was isolated from parasites using the QIAamp DNA blood kit (Qiagen). A total of 10 μg of precipitated PM1KO DNA, 1B2, and 1B11 DNA and 10 ng of pGRP170‐DDD plasmid were digested overnight with Mfe1 (New England Biolabs). The biotinylated probe used was generated by PCR using biotinylated‐16‐UTP (Sigma) and primers P3 and P4. The biotinylated probe on the southern blot was detected using IRDye 800CW streptavidin‐conjugated dye (LICOR Biosciences) and imaged using the Odyssey infrared imaging system (LICOR Biosciences).

### 4.4 Growth assays using flow cytometry

TMP was removed from asynchronous PfGRP170‐GFP‐DDD cultures for growth assays by washing the culture in equal volume of complete RPMI three times. The culture was then resuspended in complete RMPI media containing either 2.5 μg ml^−1^ blasticidin (Sigma) for conditional inhibition (Sigma) or 2.5 μg ml^−1^ blasticidin (Sigma) and 20 μM TMP (Sigma) for the control. Parasitemia was monitored using a flow cytometer, either a CyAn ADP (Beckman Coulter) or CytoFLEX (Beckman Coulter) instrument, using either 1.5 μg ml^−1^ acridine orange (Molecular Probes) as described previously (Cobb et al., 2017) or similarly using 8 μM Hoechst in filtered 1X phosphate‐buffered saline (PBS). Flow cytometry data were analysed using FlowJo software (Treestar Inc.). If the parasitemia was too high, parasites were subcultured during the experiment, and the relative parasitemia was then calculated by multiplying the calculated parasitemia by the dilution factor. Parasitemia was normalised by using the highest parasitemia as 100%. Using Prism software (GraphPad Software Inc.), the parasitemia data were fit to an exponential growth curve equation.

To determine the EC_50_ of TMP for PfGRP170‐GFP‐DDD cell lines, parasites were washed as described above and seeded into a 96‐well plate with 2.5 μg ml^−1^ blasticidin and varying TMP concentrations. Parasitemia was measured after 48 hr using flow cytometry as described above. The parasitemia data were fit to a dose–response equation using Prism.

For the IPP rescue experiment, asynchronous PfGRP170‐GFP‐DDD parasites were washed as described above and resuspended in media either with 2.5 μg ml^−1^ blasticidin or 2.5 μg ml^−1^ blasticidin and 20 μM TMP with or without 200 μM Isopentenyl pyrophosphate (Isoprenoids LC). Parasitemia were monitored using flow cytometry as described above and the data were fit to an exponential growth curve equation using Prism.

For the heat shock experiment, asynchronous PfGRP170‐GFP‐DDD parasites were washed as described above and resuspended in media either with 2.5 μg ml^−1^ blasticidin or 2.5 μg ml^−1^ blasticidin and 20 μM TMP. Parasites were then incubated at either 37°C or 40°C for 6 hr. After 6 hr, 20 μM TMP was added to cultures that were incubated without it, and all parasites were shifted back to 37°C. Parasitemia was monitored using flow cytometry as described above and the data were fit to an exponential growth curve equation (GraphPad Software Inc.).

For growth assays done with PfGRP170‐GFP‐DDD‐GFP cell lines overexpressing PfBiP, asynchronous parasites were washed as described above and resuspended in media either with 2.5 μg ml^−1^ blasticidin and 250 nM of DSM1 or 2.5 μg ml^−1^ blasticidin, 250 nM of DSM1, and 20 μM TMP. Parasitemia were monitored using flow cytometry as described above, and the data were fit to an exponential growth curve equation using Prism.

### 4.5 Synchronised growth assay

PfGRP170‐GFP‐DDD parasites were synchronised as described previously by sorbitol (VWR), followed by percoll (Genesee Scientific) the next day and then sorbitol 4 hr later to obtain 0–4 hr rings (Florentin et al., 2017; Muralidharan et al., 2011). Parasites were washed as described above to remove TMP from the media and incubated in media either with 2.5 μg ml^−1^ blasticidin or 2.5 μg ml^−1^ blasticidin and 20 μM TMP. Thin blood smears using the Hema 3 Staining Kit (PROTOCOL/Fisher) were prepared every few hours to monitor parasite growth and morphology. Slides were imaged using a Nikon Eclipse E400 microscope with a Nikon DS‐L1‐5M imaging camera.

### 4.6 Western blot

Western blotting was performed as described previously (Florentin et al., 2017). Parasite pellets were isolated using cold 0.04% Saponin (Sigma) in 1X PBS for 10 min as described previously (Florentin et al., 2017; Muralidharan et al., 2011). Antibodies used for this study were the following: mouse anti‐GFP JL‐8 (Clontech, 1:3000), rabbit anti‐PfEF1α (from D. Goldberg, 1:2,000), mouse anti‐plasmepsin V (from D. Goldberg, 1:400), rabbit anti‐PfBiP MRA‐1246 (BEI resources, 1:500), rabbit anti‐GFP A‐6455 (Invitrogen, 1:2,000), mouse anti‐eIF2α L57A5 (Cell Signalling, 1:1,000), rabbit anti‐Phospho‐eIF2α 119A11 (Cell Signalling, 1:1,000), rat anti‐HA (Roche 3F10, 1:3000), mouse anti‐Ty1 (Sigma Clone BB2, 1:1000), and mouse anti‐Ub P4D1 (Santa Cruz Biotechnology, 1:1,000). Secondary antibodies used were IRDye 680CW goat anti‐rabbit IgG and IRDye 800CW goat anti‐mouse IgG (LICOR Biosciences, 1:20,000). The western blots were imaged using the Odyssey infrared imaging system. Polyacrylamide gels used in this study were either prepared using 10% EZ‐Run protein gel solution (Fisher) or precast gradient gels (4–20%, from Bio‐Rad). Any quantification performed on western blots was done using ImageJ software. The quantification data were analysed using Prism (GraphPad Software, Inc.).

### 4.7 PK4 inhibitor experiments

Synchronised ring stage PfGRP170‐GFP‐DDD parasites were incubated in media with either 2.5 μg ml^−1^ blasticidin or 2.5 μg ml^−1^ blasticidin and 20 μM TMP in the presence or absence of a PK4 inhibitor GSK2606414 (Millipore Sigma) at 2 μM for 24 hr. After 24 hr, the parasites were lysed for western blot analysis using 0.04% saponin in 1X PBS as described above. PM1 (parental control parasites) were incubated in media with either complete RPM1 (no drug) or media containing 20 μM TMP in the presence or absence of PK4 inhibitor GSK2606414 (Millipore Sigma) at 2 μM for 24 hr. After 24 hr, the parasites were lysed for western blot analysis using 0.04% saponin in 1X PBS as described above.

### 4.8 Live fluorescence microscopy

To visualise PfGRP170‐GFP‐DDD live parasites, 100 μl of parasite culture was pelleted. The supernatant was removed, and the parasites were resuspended in 100 μl medium with 2.5 μg ml^−1^ blasticidin and 20 μM TMP and 5 μM Hoechst. The parasites were incubated at 37°C for 20 min. The parasites were then pelleted again, and 90% of the medium was removed. Parasites were resuspended in the remaining medium, and 8 μl of this culture was placed on a glass slide and covered with a coverslip. The edges were sealed with nail polish, and the cells were imaged using a DeltaVision II microscope.

### 4.9 Immunofluorescence trafficking assays and imaging processing

IFAs were performed as described previously using a combination of 4% paraformaldehyde and 0.015% glutaraldehyde for fixation and permeabilisation using 0.1% Triton‐X100 (Cobb et al., 2017; Florentin et al., 2017) or by smearing cells on a slide and fixing them with acetone. For apicoplast and RBC trafficking assays, cells were synchronised, and TMP was removed as described above. Cells were then fixed as described above, 24 hr after the removal of TMP.

Primary antibodies used for IFAs in this study were the following: rabbit anti‐GFP A‐6455 (Invitrogen, 1:200), rat anti‐PfBiP MRA‐1247 (BEI resources, 1:125), rabbit anti‐PfBiP MRA‐1246 (BEI resources (1:100), mouse anti‐plasmepsin V (from D. Goldberg, 1:1), mouse anti‐GFP clones 7.1 and 13.1 (Roche 11814460001, 1:500), rabbit anti‐Cpn60 (from B. Striepen, 1:1,000), rabbit anti‐PfERD2 (MR4, 1:2,000), rabbit anti‐HA 9110 (Abcam, 1:200), rabbit anti‐PfMAHRP1C (from Hans‐Peter Beck, 1:500), mouse anti‐PfFIKK4.2 (from David Cavanagh/EMRR, 1:1,000), mouse anti‐Ty1 (Sigma Clone BB2, 1:200), and rabbit anti‐PfHSP70X (from Jude Przyborski, 1:500). Secondary antibodies used in this study are Alexa Fluor goat anti‐rabbit 488, Alexa Fluor goat anti‐rabbit 546, Alexa Fluor goat anti‐mouse 488, Alexa Fluor goat anti‐mouse 546, and Alexa Fluor goat anti‐rat 546 (Life Technologies, 1:100). The mouse anti‐PfFIKK4.2, rabbit anti‐PfHSP70X, and anti‐PfMAHRP1C require acetone fixation.

All fixed cells were mounted using ProLong Diamond with DAPI (Invitrogen) and imaged using the DeltaVision II microscope system or Zeiss ELYRA S1 (SR‐SIM) super resolution microscope using a 100× objective. Images taken using the DeltaVision II were collected as a Z‐stack and were deconvolved using the DeltaVision II software (SoftWorx). The deconvolved Z‐stacks were then displayed as a maximum intensity projection using SoftWorx. Images taken using the super resolution microscope were taken as a Z‐stack. The Z‐stacks were analysed using Zen Software (Zeiss, version from 2011) for SIM processing and obtaining the maximum intensity projection. Any adjustments made to the brightness and/or contrast of the images were made using either Softworx, Zen Software, or Adobe Photoshop and were done for display purposes only. Any quantification performed for microscopy images was done using ImageJ software as described previously (Cobb et al., 2017). The quantification data were analysed using Prism (GraphPad Software, Inc.).

### 4.10 Co‐IP assays and mass spectroscopy

Parasites pellets were isolated from 48 ml of asynchronous culture at high parasitemia (10% or higher) using cold 0.04% saponin in 1X PBS as described above. Parasite pellets were lysed by resuspending the pellet in 150 μl of extraction buffer (40 mM Tris HCL pH 7.6, 150 mM KCL, and 1 mM EDTA) with 0.5% NP‐40 (VWR) and 1X HALT protease inhibitor (Thermo). The resuspended parasites were then incubated on ice for 15 min and then sonicated three times (10% amplitude, 5‐s pulses). In between each sonication, the lysate was placed on ice for 1 min. The lysate was then centrifuged at 21,100 g for 15 min at 4°C. The supernatant was collected in a fresh tube and placed on ice. The remaining pellet was subjected to a second lysis step using 150 μl of extraction buffer as above without NP‐40. The lysate was sonicated and centrifuged as above (no 15‐min incubation on ice). The supernatant was collected and combined with the lysate from the first lysis step (INPUT sample). A 20 μl of the input sample was collected into a fresh tube and stored in the −80°C. The remaining input sample was combined with 2 μl of rabbit anti‐GFP monoclonal G10362 (Thermo) and incubated rocking for 2 hr at 4°C.

After the 2‐hr incubation, the lysate with antibody was added to 50 μl of prepared protein G Dynabeads (Invitrogen). Dynabeads were prepared by washing 50 μl of beads three times with 100 μl of IgG binding buffer (20 mM Tris HCL pH 7.6, 150 mM KCL, 1 mM EDTA, and 0.1% NP‐40). The IgG binding buffer was removed from the beads each time using a magnetic rack (Life Technologies). The beads, antibody, and lysate were incubated rocking for 2 hr at 4°C. After the 2‐hr incubation, the unbound fraction of protein was collected using the magnetic rack into a fresh tube and stored at −80°C until needed for western blot analysis. The beads were then washed two times in 300 μl of IgG binding buffer with 1X HALT and one time in IgG binding buffer with 1X HALT without NP‐40. Each wash was done for 10 min rocking at 4°C.

For co‐IP's to show PfGRP170‐GFP‐DDD/BiP interaction 0–4 hr ring stage parasites were obtained, and TMP was removed as described under the synchronised growth assay section. Parasites were lysed and an anti‐GFP IP was performed as described above, approximately 24 hr after the removal of TMP. Protein was eluted off the beads for western blot using 1X Protein Loading Dye (LICOR) with 2.5% beta‐Mercaptoethanol (Fisher) and boiled for 5 min. This was followed by a centrifugation at 16,200 g for 5 min. The eluted proteins are collected by placing the tube on a magnetic rack. The isolated proteins on magnetic beads were digested with trypsin and analysed at the Emory University Integrated Proteomics Core using a Fusion Orbitrap mass spectrometer.

### 4.11 PfGRP170‐BirA biotinylation and mass spectrometry

To confirm that proteins were biotinylated when biotin was added to the PfGRP170‐BirA parasites, parasites were incubated 24 hr in media containing 2.5 nM WR + 150 μg of biotin (Sigma). Parasites were isolated using 0.04% saponin in 1X PBS, and the lysates were analysed via western blot as described above. Secondary antibodies used were IRDye 680CW goat anti‐rabbit IgG and IRDye 800CW Streptavidin (LICOR). 3D7 parasites incubated with media containing 150 μg of biotin for 24 hr was used as a control.

For PfGRP170‐HA‐BirA streptavidin IP's, cultures were incubated for 24 hr in media containing 2.5 nM WR + 150 μg of biotin (Sigma). A total of 48 ml of asynchronous culture at high parasitemia (10% or higher) were harvested for IP as described above with the following modifications. Streptavidin MagneSphere Paramagnetic Particle beads (Promega) were used to isolated biotinylated proteins. To prepare the Streptavidin beads for IP, beads were washed three times in 1 ml of 1X PBS. Incubations of lysate with the magnetic beads were performed at room temperature for 30 min. After the unbound fraction was removed, beads were washed twice in 8 M Urea (150 mM NaCL, 50 mM Tris HCL pH 7.4) and once in 1X PBS. The biotinylated proteins on magnetic beads were digested with trypsin and analysed at the Emory University Integrated Proteomics Core using a Fusion Orbitrap Mass Spectrometer. 3D7 control streptavidin IP's were conducted as above but without the addition of 2.5 nM WR to the media.

### 4.12 Proximity ligation assays

Asynchronous PfGRP170‐GFP‐DDD parasites were fixed as described above, approximately 24 hr after the removal of TMP. The PLA was performed using the Duolink PLA Fluorescence kit (Sigma) per the manufacturers protocol. For the BiP/PfGRP170 PLA assay, primary antibodies mouse anti‐GFP (Roche 11814460001, 1:500) and rabbit anti‐BiP MRA‐1246 (BEI resources (1:100) were used. For the negative control primary antibodies mouse anti‐plasmepsin V (from D. Goldberg, 1:1) and rabbit anti‐GFP A‐6455 (Invitrogen, 1:200) were used.

### 4.13 Ring stage survival assay

The ring‐stage survival assay method was performed on 3D7 (control) and PfGRP170‐BirA parasites as described previously, with a slight adjustment (Witkowski et al., 2013). Cultures were synchronised using 5% sorbitol (Sigma‐Aldrich, St. Louis, MO, USA), prewarmed to 37°C, to obtain the highest proportion of rings, ≥50%. The cultures were placed back under previously described conditions for 24 hr and followed‐up the next morning. Thin blood smears were methanol fixed and stained with 10% Giemsa for 15 min and evaluated for mature schizonts with visible nuclei (Boddey & Cowman, 2013; Deponte et al., 2012; Zhang et al., 2017). The parasites were independently suspended in PRMI‐1640 supplemented with 15 U ml^−1^ of sodium heparin (Sigma‐Aldrich, St. Louis, MO, USA) to disrupt spontaneous rosettes formation for 15 min at 37°C. After incubation, each parasite culture was layered onto a 75/25% percoll (GE Healthcare Life Sciences, Pittsburgh, PA, USA) gradient and centrifuged at 3,000 rpm for 15 min. The intermediate phases containing the mature schizonts of each culture were independently collected, gently washed in RPMI, and transferred into two new T25 flasks with fresh cRPMI and erythrocytes for 3‐hr incubation at previously described conditions. Thin blood smears were prepared as previously described, to ensure >10% schizonts count.

At the 3‐hr mark, the parasites were taken‐out of incubation and treated with 5% sorbitol to remove the remaining mature schizonts, which had not invaded erythrocytes yet. Parasitemia was adjusted to 1% at 2% haematocrit by adding uninfected erythrocytes and cPRMI, after the evaluation of quick stained Giemsa smears. The parasites were exposed to 700 nM DHA or 1% dimethyl sulfoxide (DMSO) for 6 hr. After the 6‐hr incubation period, the parasites were washed to remove the drug or DMSO and resuspended in 1 ml of cRPMI. The parasites were then transferred into two new well in the 48‐well culture plate, incubated at 37°C under a 90% N_2_, 5% CO_2_, and 5% O_2_ gas mixture for 66 hr, after which thin blood smears were prepared, methanol fixed, stained with 10% Giemsa for 15 min and read by three operators. Growth rate and percent survival were calculated by counting the number of parasitised cells in an estimated 2,000 erythrocytes**.**

## Supporting information

Figure S1. Sequence Alignment of Lhs1 and PfGRP170
Sequence alignment of S. cerevisiae GRP170 (Lhs1) and PfGRP170. The alignment was performed using EMBOSS Needle which creates a global alignment of two sequences using the Needleman‐Wunsch algorithm. The software used to do this is provided by the European Bioinformatics Institute, which is a part of the European Molecular Biology Laboratory (EMBL). Identical residues are indicated by a “I”, strongly similar residues are indicated by a “:”, and weakly similar residues are indicated by a “.”.

Figure S2. Sequence homology of PfGRP170
Sequence identify and homology of P. falciparum GRP170 compared to GRP170 homologs from other Plasmodium Species (P. vivax (PVX_083105), P. malariae (PmUG01_12020700), P. ovale (PocGH01_12018900), and P. berghei (PBANKA_1357200)), T. gondii GRP170 (TGGT1_226830), yeast GRP170 (S. cerevisiae), and human GRP170 (H. sapiens). Alignments to determine sequence identify and homology were performed using EMBOSS Needle which creates a global alignment of two sequences using the Needleman‐Wunsch algorithm. The software to do this is provided by the European Bioinformatics Institute, which is a part of the European Molecular Biology Laboratory (EMBL).

Figure S3. PfGRP170 is Expressed Throughout the Asexual Life Cycle
TMP was removed from tightly synchronized ring stage PfGRP170‐GFP‐DDD parasites and protein was isolated throughout the asexual life cycle. Lysates were separated on a Western blot and probed with anti‐GFP to visualize PfGRP170‐GFP‐DDD and anti‐PfEF1α as a loading control.

Figure S4. Conditional mutants of PfGRP170 localize to the ER
Synchronized PfGRP170‐GFP‐DDD ring stage parasites were incubated with and without TMP for 24 hours. Parasites were then fixed with paraformaldehyde and stained with either DAPI, anti‐GFP, and anti‐BiP (ER) **(A)** or DAPI, anti‐GFP, and anti‐ERD2 (Golgi) **(B).** Images were taken as a Z‐stack using super resolution microscopy and SIM processing was performed on the Z‐stacks. Images are displayed as a maximum intensity projection. The scale bar is 2 μm.

Figure S5. PfGRP170‐BirA localizes to the parasite ER and biotinylates proteins.
**(A).** Western blot of 3D7 (parental) and PfGRP170‐BirA expressing parasites probed with anti‐HA and anti‐EF1α.
**(B).** Paraformaldehyde fixed PfGRP170‐BirA parasites stained with anti‐HA (PfGRP170‐BirA), anti‐PfPMV (ER), and DAPI. The images were taken with Delta Vision II, deconvolved and are displayed as a maximum intensity projection. The scale bar is 5 μm.
**(C).** A western blot analysis of 3D7 (parental) and PfGRP170‐BirA parasites following a 24‐hour incubation with biotin is shown. A fluorophore‐labeled streptavidin secondary antibody was used to visualize biotinylated proteins. A control with PfGRP170‐BirA parasites incubated without biotin is also shown. Anti‐EF1α is used as a loading control.

Figure S6. Relative transcript abundance of proteins identified in both the anti‐GFP co‐immunoprecipitation and BioID mass spectroscopy approaches
The relative transcript abundance of the 11 PfGRP170 interacting proteins identified in Figure 4. The data are plotted using previously published genome‐wide real‐time transcription data^46^.

Figure S7. PfGRP170 is not Required for Trafficking to the Host RBC.
Tightly synchronized ring stage PfGRP170‐GFP‐DDD parasites were incubated with and without TMP for 24 hours. Following this incubation, parasites were fixed with acetone and stained with DAPI, anti‐GFP (PfGRP170) and either anti‐PfFIKK4.2 **(A),** anti‐PfMAHRP1C **(B),** or anti‐PfHSP70X **(C).** The images were taken with Delta Vision II, deconvolved, and are displayed as a maximum intensity projection. The scale bar is 5 μM. Mean Fluorescent Intensity (M.F.I) was calculated for the exported fraction (PfFIKK4.2, PfMAHRP1C, and PfHSP70x) from individual cells. Data are from two independent experiments and is displayed as box‐and‐whiskers plots (whiskers represent the maximum and minimum M.F.I). The significance was calculated using an unpaired t test (NS = not significant).

Figure S8. Overexpression of PfGRP170 does not Confer Artemisinin Resistance
Tightly synchronized ring stage 3D7 and PfGRP170‐BirA parasites were incubated with either 1% DMSO (Control) or Dihydroartemsinin (DHA) for 6 hours. After 6 hours the drug is removed by washing the culture with complete RPMI. Parasitemia was calculated using Giemsa stained thin blood smears at 0 hours (to calculate starting parasitemia) and 72 hours after either DMSO or DHA exposure. Four independent replicates of the experiment were completed for 3D7 and three for PfGRP170‐BirA. The growth rate of the 3D7 and PfGRP170‐BirA parasites, incubated only with DMSO, was calculated after 72 hours **(A).** The percent survival of parasites was calculated for 3D7 and PfGRP170‐BirA after DHA exposure was calculated after 72 hours **(B).**

Figure S9. EIF2‐α levels do not change in PM1 parasites in the presence or absence of TMP or a PK4 inhibitor.
**(Top)** Synchronized ring stage PM1 parasites were incubated with and without TMP and in the presence and absence of 2 μM PK4 inhibitor GSK2606414 for 24 hours. Protein was isolated from these samples and analyzed via western blot by probing for anti‐eIF2a and anti‐Phospho‐eIF2α. **(Bottom)** The ratio of phosphorylated EIF2α over total EIF2α in PM1 parasites incubated with and without TMP is shown. Western blot band intensities were calculated using ImageJ software (NIH) and the significance was calculated using an unpaired t test. Data are representative of 2 biological replicates ± S.E.M.

Table S1. Raw Mass Spectroscopy data
Raw mass spectroscopy data from two anti‐GFP IP (using 1B2 and 1B11), two parental anti‐GFP IP's (using PM1), two Streptavidin IP's the PfGRP170‐BirA cell lines following a 24 incubation with biotin, and one streptavidin IP's of 3D7 cell lines following a 24 hour incubation with biotin. Both approaches used asynchronous cells. The excel file includes the following: A total list of proteins from PlasmoDB containing a signal peptide and/or transmembrane domain used to sort the mass spectroscopy data (Tab 1), raw mass spectroscopy data from PM1 anti‐GFP IP's 1 and 2 (Tabs 2 and 3), list of proteins from the PM1 anti‐GFP IP's 1 and 2 that contained a signal peptide and/or transmembrane domain (Tabs 4 and 5), raw mass spectroscopy data from the 1B2 anti‐GFP IP (Tab 6) and the list of proteins from the 1B2 anti‐GFP IP that contained a signal peptide and/or transmembrane domain (Tab 7), raw mass spectroscopy data from the 1B11 anti‐GFP IP (Tab 8) and the list of proteins from the 1B11 anti‐GFP IP that contained a signal peptide and/or transmembrane domain (Tab 9), raw mass spectroscopy data from a streptavidin IP on 3D7 parasites incubated with biotin (Tab 10), the list of proteins from the 3D7 streptavidin IP that contained a signal peptide and/or transmembrane domain (Tab 11), raw mass spectroscopy data from two independent PfGRP170‐BirA streptavidin IP's (Tabs 12 and 13) and the list of proteins from the PfGRP170‐BirA streptavidin IP's that contained a signal peptide and/or transmembrane domain (Tabs 14 and 15).

Table S2. Primers used in this study
