# Supplementary figures and images for: The endoplasmic reticulum chaperone PfGRP170 is essential for asexual development and is linked to stress response in malaria parasites
Source: Cell Microbiol. 2019 Jun 6;21(9):e13042. doi: 10.1111/cmi.13042 (PMC6699899; doi:10.1111/cmi.13042)

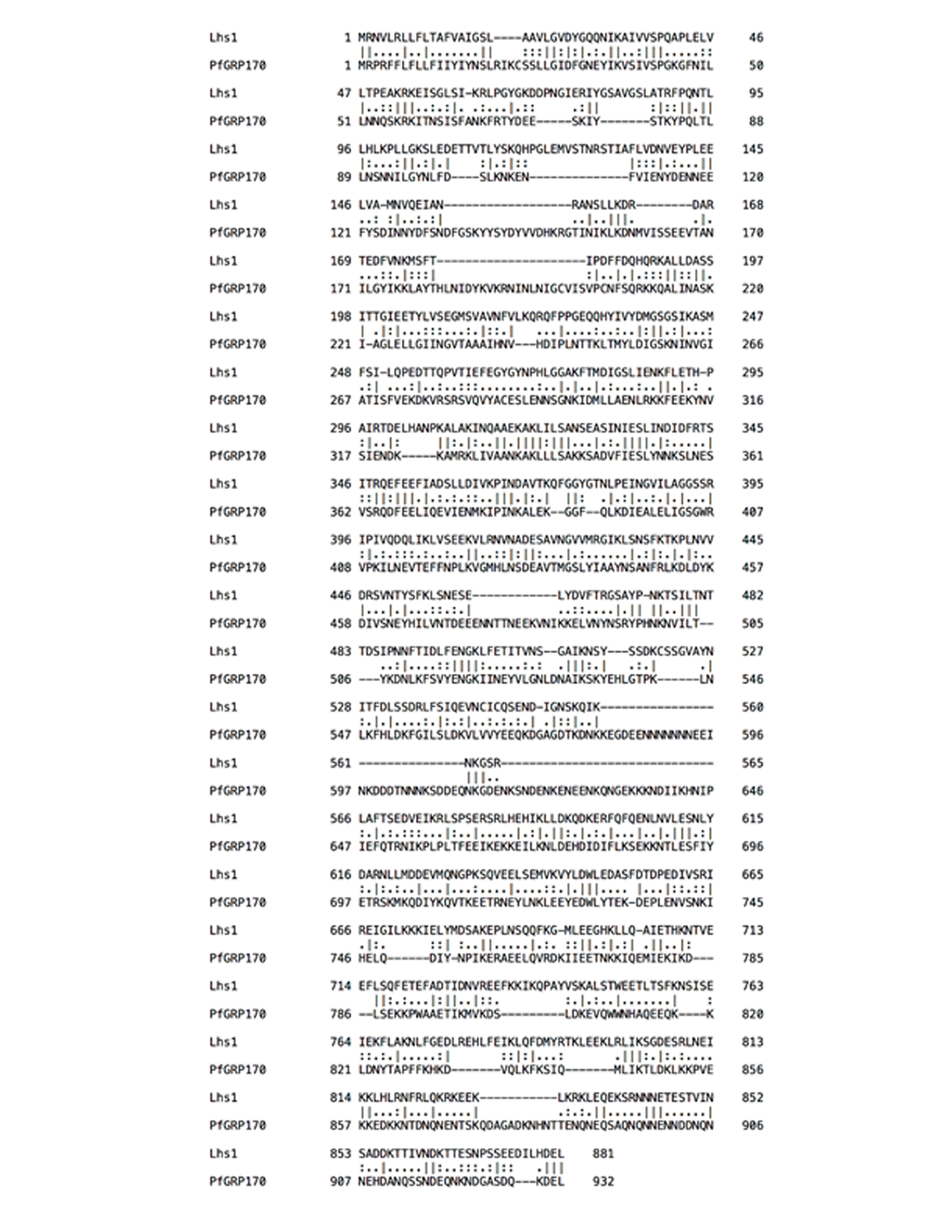

Supplement: Supplementary file 1 — Figure S1. Sequence Alignment of Lhs1 and PfGRP170 Sequence alignment of S. cerevisiae GRP170 (Lhs1) and PfGRP170. The alignment was performed using EMBOSS Needle which creates a global alignment of two sequences using the Needleman‐Wunsch algorithm. The software used to do this is provided by the European Bioinformatics Institute, which is a part of the European Molecular Biology Laboratory (EMBL). Identical residues are indicated by a “I”, strongly similar residues are indicated by a “:”, and weakly similar residues are indicated by a “.”. [file CMI-21-na-s001.tif]

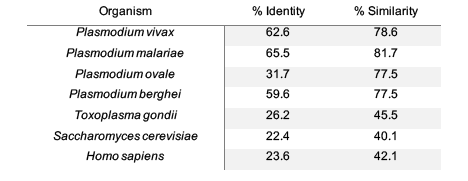

Supplement: Supplementary file 2 — Figure S2. Sequence homology of PfGRP170 Sequence identify and homology of P. falciparum GRP170 compared to GRP170 homologs from other Plasmodium Species (P. vivax (PVX_083105), P. malariae (PmUG01_12020700), P. ovale (PocGH01_12018900), and P. berghei (PBANKA_1357200)), T. gondii GRP170 (TGGT1_226830), yeast GRP170 (S. cerevisiae), and human GRP170 (H. sapiens). Alignments to determine sequence identify and homology were performed using EMBOSS Needle which creates a global alignment of two sequences using the Needleman‐Wunsch algorithm. The software to do this is provided by the European Bioinformatics Institute, which is a part of the European Molecular Biology Laboratory (EMBL). [file CMI-21-na-s002.tif]

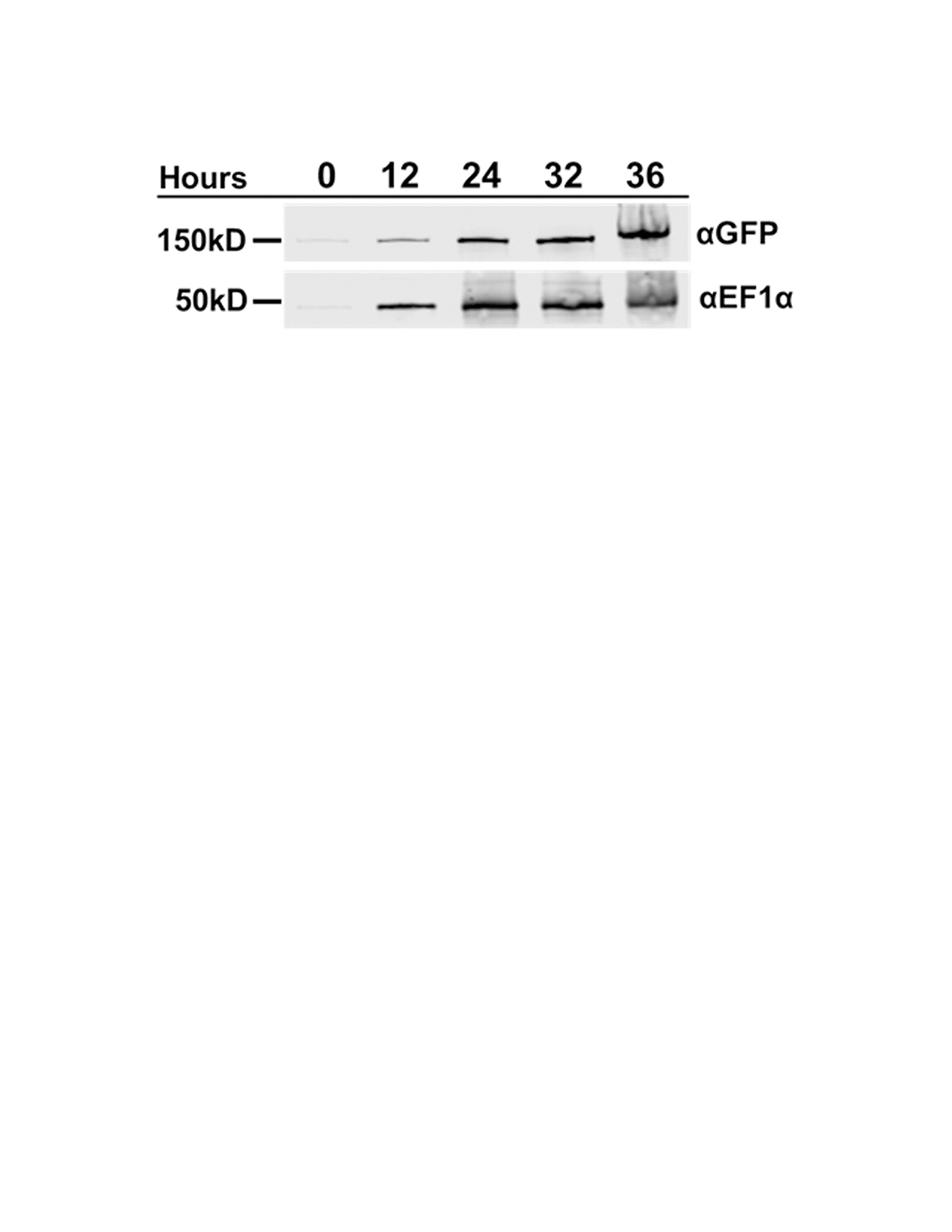

Supplement: Supplementary file 3 — Figure S3. PfGRP170 is Expressed Throughout the Asexual Life Cycle TMP was removed from tightly synchronized ring stage PfGRP170‐GFP‐DDD parasites and protein was isolated throughout the asexual life cycle. Lysates were separated on a Western blot and probed with anti‐GFP to visualize PfGRP170‐GFP‐DDD and anti‐PfEF1α as a loading control. [file CMI-21-na-s003.tif]

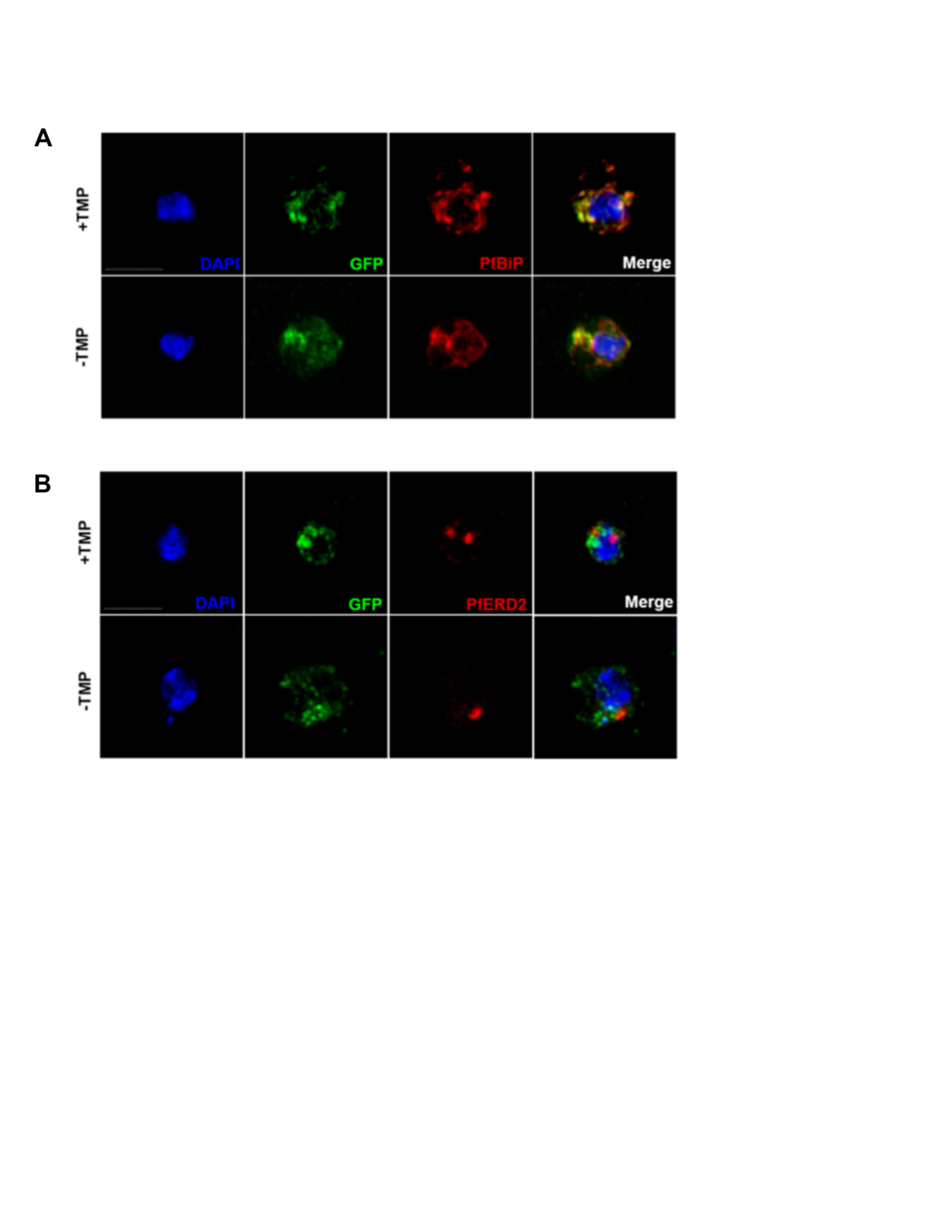

Supplement: Supplementary file 4 — Figure S4. Conditional mutants of PfGRP170 localize to the ER Synchronized PfGRP170‐GFP‐DDD ring stage parasites were incubated with and without TMP for 24 hours. Parasites were then fixed with paraformaldehyde and stained with either DAPI, anti‐GFP, and anti‐BiP (ER) (A) or DAPI, anti‐GFP, and anti‐ERD2 (Golgi) (B). Images were taken as a Z‐stack using super resolution microscopy and SIM processing was performed on the Z‐stacks. Images are displayed as a maximum intensity projection. The scale bar is 2 μm. [file CMI-21-na-s004.tif]

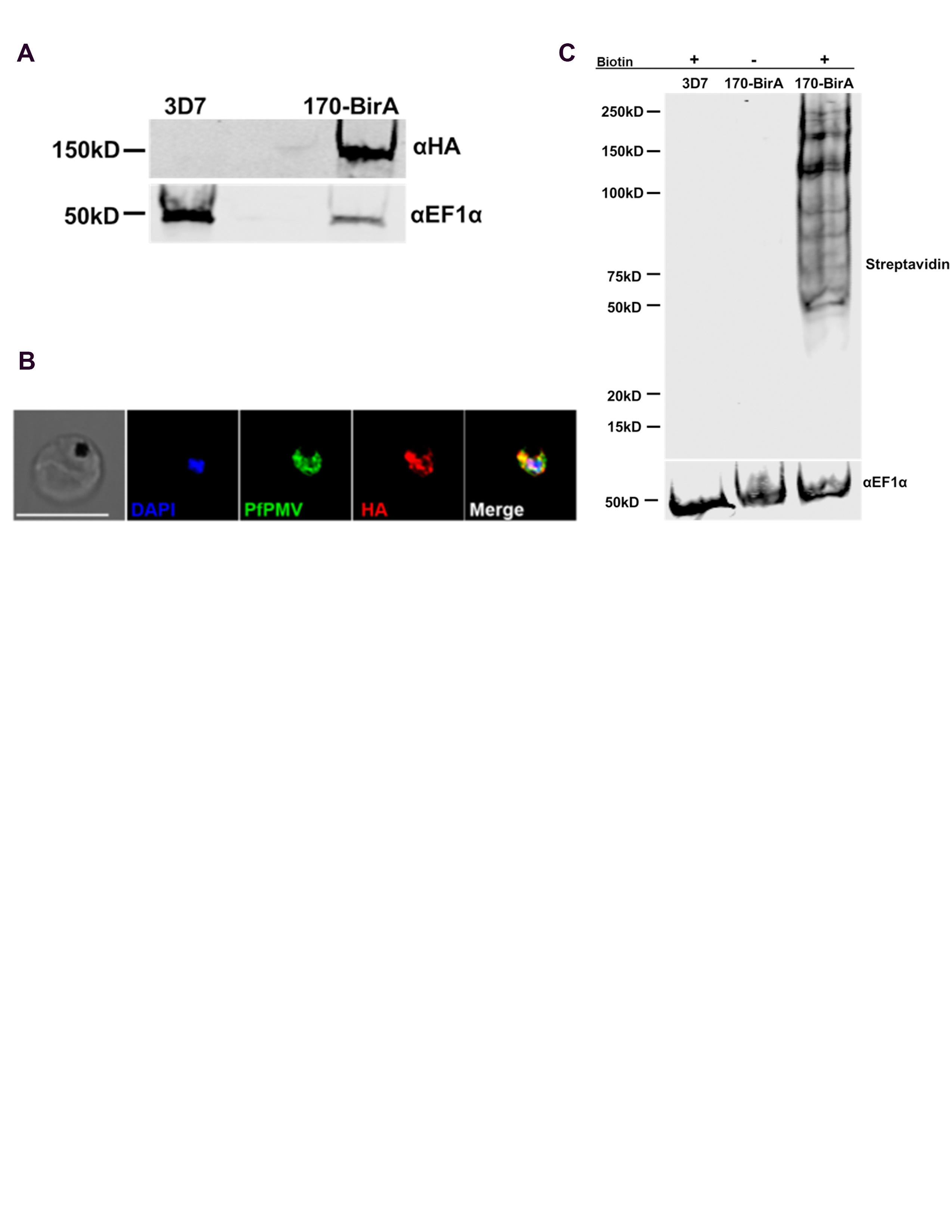

Supplement: Supplementary file 5 — Figure S5. PfGRP170‐BirA localizes to the parasite ER and biotinylates proteins. (A). Western blot of 3D7 (parental) and PfGRP170‐BirA expressing parasites probed with anti‐HA and anti‐EF1α. (B). Paraformaldehyde fixed PfGRP170‐BirA parasites stained with anti‐HA (PfGRP170‐BirA), anti‐PfPMV (ER), and DAPI. The images were taken with Delta Vision II, deconvolved and are displayed as a maximum intensity projection. The scale bar is 5 μm. (C). A western blot analysis of 3D7 (parental) and PfGRP170‐BirA parasites following a 24‐hour incubation with biotin is shown. A fluorophore‐labeled streptavidin secondary antibody was used to visualize biotinylated proteins. A control with PfGRP170‐BirA parasites incubated without biotin is also shown. Anti‐EF1α is used as a loading control. [file CMI-21-na-s005.tif]

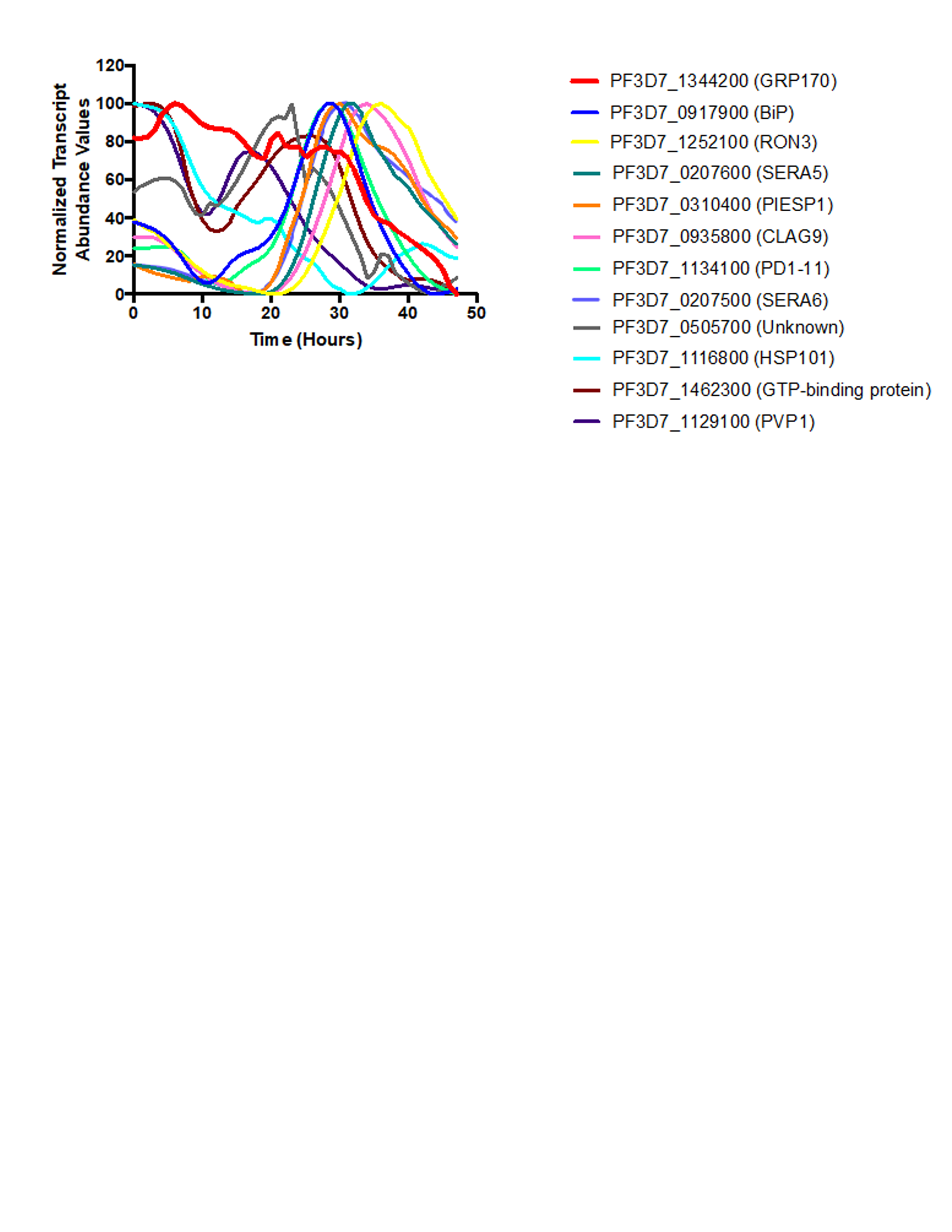

Supplement: Supplementary file 6 — Figure S6. Relative transcript abundance of proteins identified in both the anti‐GFP co‐immunoprecipitation and BioID mass spectroscopy approaches The relative transcript abundance of the 11 PfGRP170 interacting proteins identified in Figure 4. The data are plotted using previously published genome‐wide real‐time transcription data46. [file CMI-21-na-s006.tif]

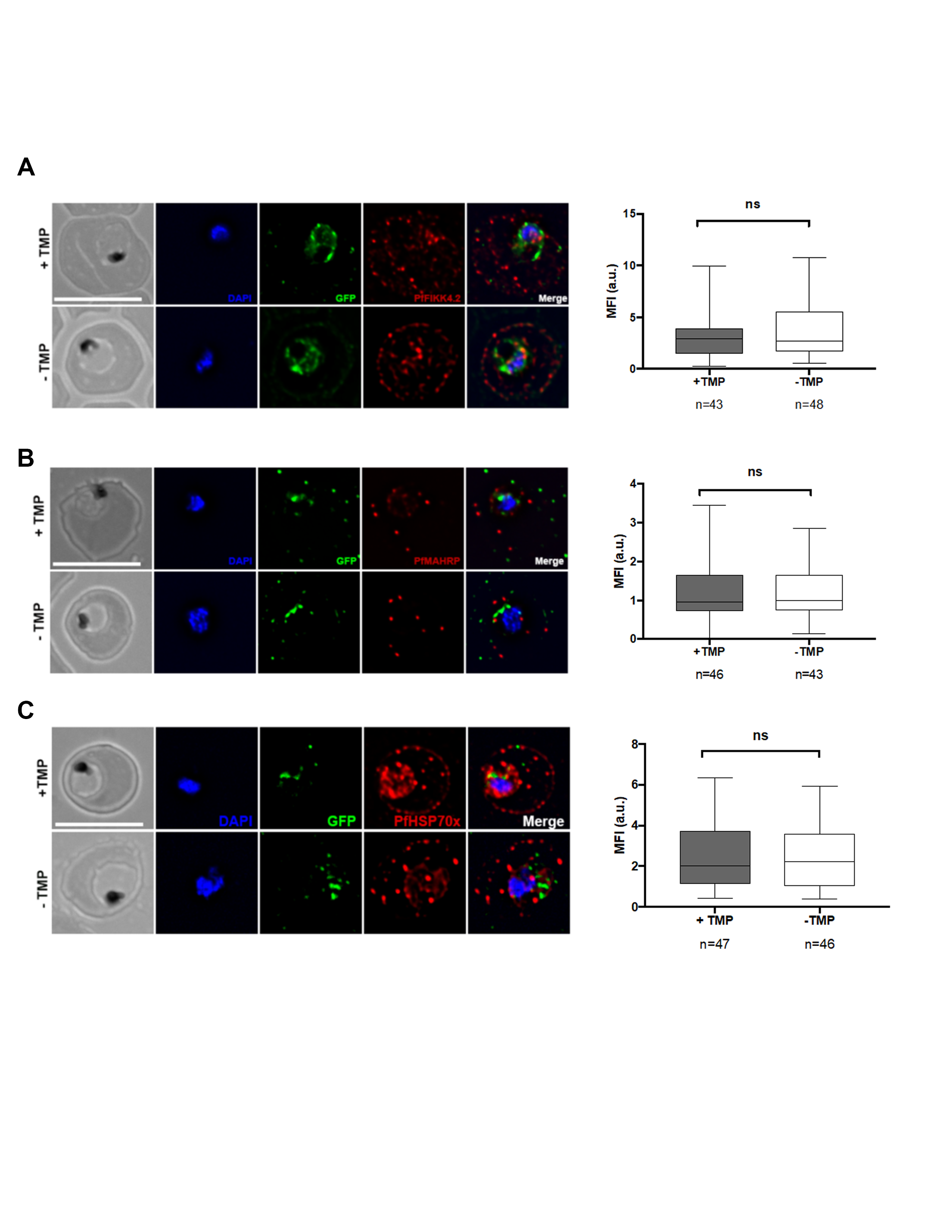

Supplement: Supplementary file 7 — Figure S7. PfGRP170 is not Required for Trafficking to the Host RBC. Tightly synchronized ring stage PfGRP170‐GFP‐DDD parasites were incubated with and without TMP for 24 hours. Following this incubation, parasites were fixed with acetone and stained with DAPI, anti‐GFP (PfGRP170) and either anti‐PfFIKK4.2 (A), anti‐PfMAHRP1C (B), or anti‐PfHSP70X (C). The images were taken with Delta Vision II, deconvolved, and are displayed as a maximum intensity projection. The scale bar is 5 μM. Mean Fluorescent Intensity (M.F.I) was calculated for the exported fraction (PfFIKK4.2, PfMAHRP1C, and PfHSP70x) from individual cells. Data are from two independent experiments and is displayed as box‐and‐whiskers plots (whiskers represent the maximum and minimum M.F.I). The significance was calculated using an unpaired t test (NS = not significant). [file CMI-21-na-s007.tif]

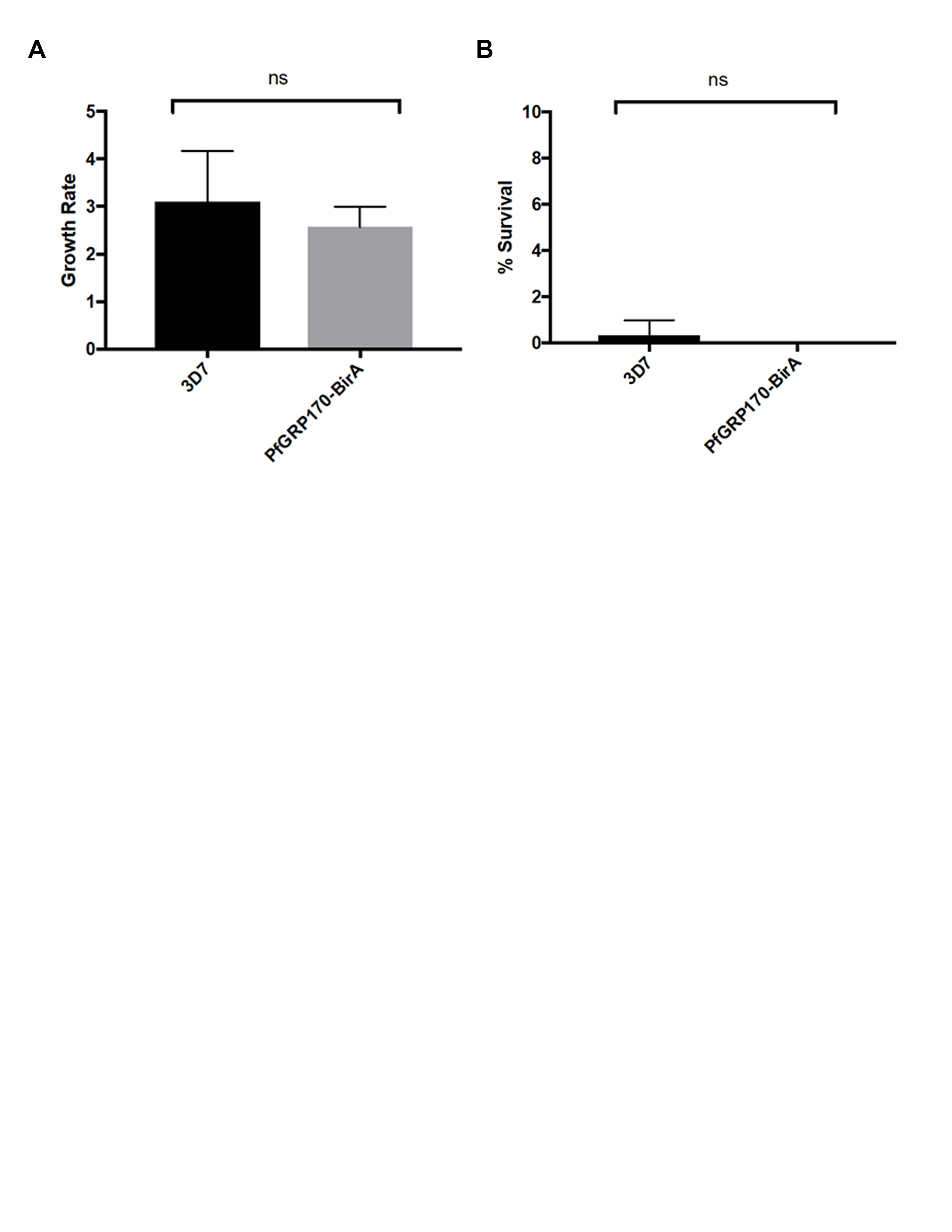

Supplement: Supplementary file 8 — Figure S8. Overexpression of PfGRP170 does not Confer Artemisinin Resistance Tightly synchronized ring stage 3D7 and PfGRP170‐BirA parasites were incubated with either 1% DMSO (Control) or Dihydroartemsinin (DHA) for 6 hours. After 6 hours the drug is removed by washing the culture with complete RPMI. Parasitemia was calculated using Giemsa stained thin blood smears at 0 hours (to calculate starting parasitemia) and 72 hours after either DMSO or DHA exposure. Four independent replicates of the experiment were completed for 3D7 and three for PfGRP170‐BirA. The growth rate of the 3D7 and PfGRP170‐BirA parasites, incubated only with DMSO, was calculated after 72 hours (A). The percent survival of parasites was calculated for 3D7 and PfGRP170‐BirA after DHA exposure was calculated after 72 hours (B). [file CMI-21-na-s008.tif]

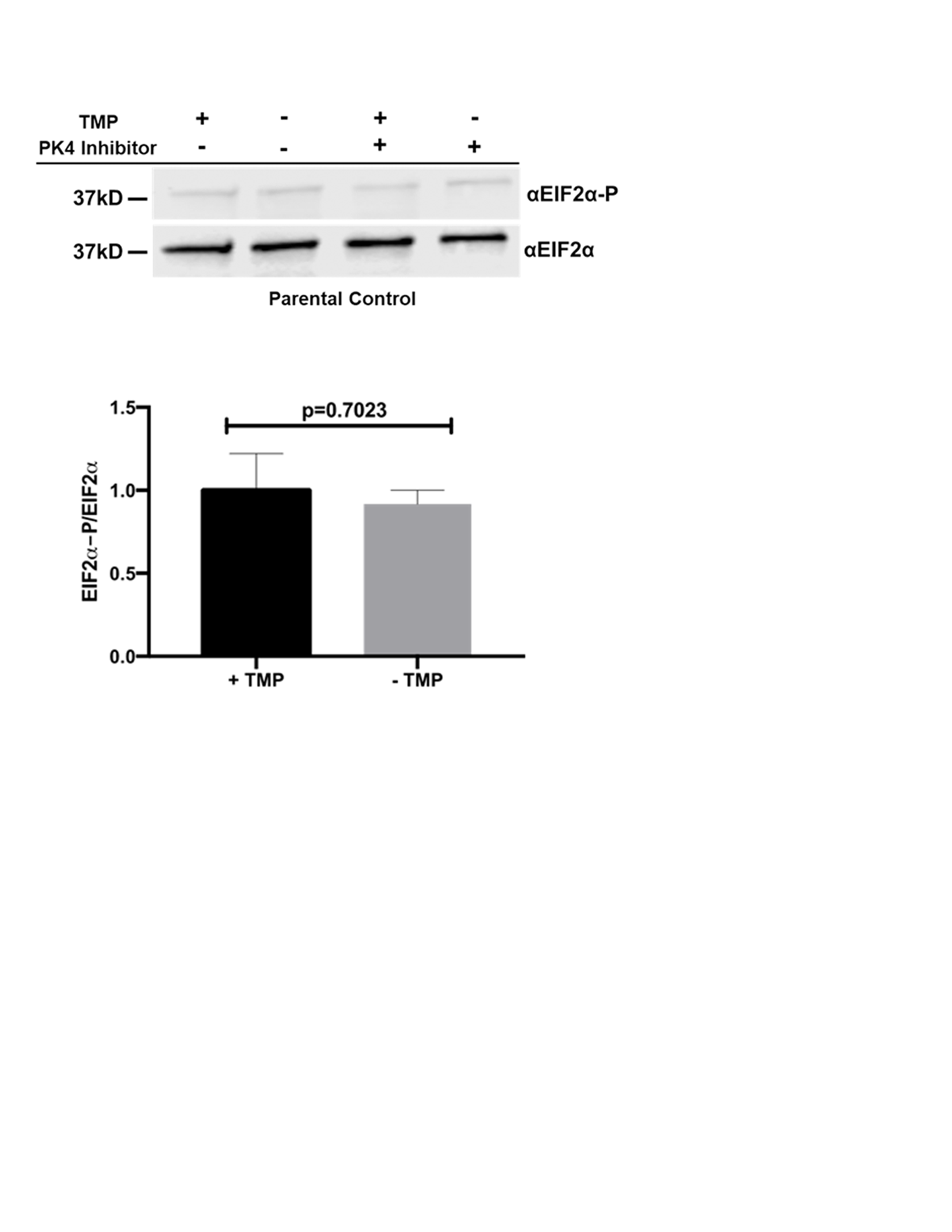

Supplement: Supplementary file 9 — Figure S9. EIF2‐α levels do not change in PM1 parasites in the presence or absence of TMP or a PK4 inhibitor. (Top) Synchronized ring stage PM1 parasites were incubated with and without TMP and in the presence and absence of 2 μM PK4 inhibitor GSK2606414 for 24 hours. Protein was isolated from these samples and analyzed via western blot by probing for anti‐eIF2a and anti‐Phospho‐eIF2α. (Bottom) The ratio of phosphorylated EIF2α over total EIF2α in PM1 parasites incubated with and without TMP is shown. Western blot band intensities were calculated using ImageJ software (NIH) and the significance was calculated using an unpaired t test. Data are representative of 2 biological replicates ± S.E.M. [file CMI-21-na-s009.tif]
