# Supplementary material for: The endoplasmic reticulum chaperone PfGRP170 is essential for asexual development and is linked to stress response in malaria parasites
Source: Cell Microbiol. 2019 Jun 6;21(9):e13042. doi: 10.1111/cmi.13042 (PMC6699899; doi:10.1111/cmi.13042)
Supplement: Supplementary file 11 — Table S2. Primers used in this study [file CMI-21-na-s011.docx]

| **Primer** | **Sequence** |
| --- | --- |
| P1 | cataaatatattatataactcgacgcggccgtcaaagttcatcactagcgtaatctggaacatcgtatggg |
| P2 | cccatacgatgttccagattacgctagtgatgaactttgacggccgcgtcgagttatataatatatttatg |
| P3 | CACTATAGAACTCGAGGATAAAGTTCTTGTTGTTTATGAAGAACAAAAAGATGGAGCTGG |
| P4 | CTGCACCTGGCCTAGGTTGATCTGATGCTCCATCATTTTTATTTTGCTCATCGTTGG |
| P5 | AAAAACTCACGCTAGCATGAGACCTCGTTTTTTTTTGTTCCTACTTTTTATAATATATATATATAATAG |
| P6 | GACGTCGTACGGGTACCTAGGTTGATCTGATGCTCCATCATTTTTATTTTGCTCATCGTTG |
| P7 | GGAGCATCAGATCAACCTAGGTACCCGTACGACGTCCCGGACTACGCTGGCTATCCCTATG |
| P8 | GAACAATAATAGATCTttataattcatctttCTTCTCTGCGCTTCTCAGGGAGATTTCTCCGCCCATCCAG |
| P9 | TCTTCTCCTTTACTGACGTCACCTCTTTTATGATCTACTACATAATCATATGAATAATAC |
| P10 | TAAAAGAGGTGACGTCAGTAAAGGAGAAGAATTATTTACTGGAGTTGTCCCAATTCTTG |
| P11 | GAACAATAATAGATCTTTATTTGTATAGTTCATCCATGCCATGTGTAATCCCAGCAGCTG |
| P12 | ATGAGACCTCGTTTTTTTTTGTTCCTACTTTTTATAATATATATATATAATAGTTTAAG |
| P13 | gtgcccattaacatcaccatctaattcaacaagaattggg |

**Supplemental Table 2:** Primers used in this study
